# High-fidelity imaging of a tumour-associated lysosomal enzyme with an acceptor engineering-boosted near-infrared fluorescent probe†

**DOI:** 10.1039/d4sc00487f

**Published:** 2024-04-16

**Authors:** Bin Feng, Feiyi Chu, Yanpeng Fang, Min Liu, Xueping Feng, Jie Dong, Fei Chen, Wenbin Zeng

**Affiliations:** a Xiangya School of Pharmaceutical Sciences, Central South University Changsha 410013 China wbzeng@hotmail.com wbzeng@csu.edu.cn; b Hunan Key Laboratory of Diagnostic and Therapeutic Drug Research for Chronic Diseases Changsha 410013 China; c Xiangya Hospital, Central South University Changsha 410013 China

## Abstract

To facilitate the understanding of the dynamic distribution and activity of lysosomal enzymes, it is highly desirable to develop high-fidelity near-infrared (NIR) activatable fluorescent probes. Here, we propose a general acceptor engineering strategy to construct NIR probes with lysosome-targeting capability. Upon isosteric replacement and additional functionalization, the β-gal-activatable probe OELyso-Gal exhibited excellent lysosome-targeting capability and favorable responsive performance to the enzyme of interest. Notably, the steric hindrance effect from acceptor engineering is modest, which renders the probe unprecedented affinity to enzymes. Upon the introduction of acceptor engineering, the lysosome-targeting probe became more sensitive to β-gal in cells and tissues, boosting the discrimination of high β-gal-expressing ovarian cancer tumours from low β-gal-expressing tissues. Furthermore, the superiority of OELyso-Gal was validated in real-time visualization of ovarian cancer in tumour-bearing mice. This elegant acceptor engineering strategy provides inspirational insights into the development of customized fluorescent probes for monitoring disease-associated biomarkers within subcellular organelles.

## Abstract 2

This work reports an acceptor engineering-boosted near-infrared fluorescent probe for high-fidelity imaging of tumour-associated β-galactosidase specifically in lysosomes and *in vivo*.

## Graphical abstract

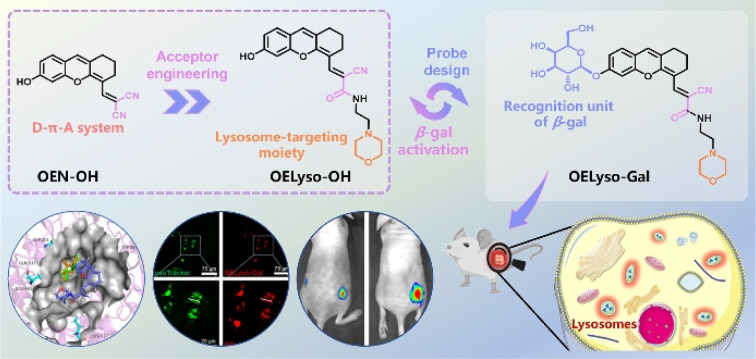

## Introduction

Currently, lysosomes are described as advanced organelles that play critical roles in cellular homeostasis and the mediation of a variety of physiological processes, such as protein degradation and plasma membrane repair.^1,2^ Pieces of evidence have shown that the aberrant activities of hydrolytic enzymes in lysosomes are associated with the pathogenesis of diseases such as storage disorders, cancer, neurodegenerative disorders, and cardiovascular diseases.^3–5^ Among these, β-galactosidase (β-gal) in lysosomes, an exoglycosidase, is involved in the catabolism of sugar conjugates, and its abnormal levels have been associated with the occurrence and progression of primary ovarian cancers, making the lysosomal enzyme a reliable biomarker for the early diagnosis and dynamic imaging of cancer.^6–10^ On-site monitoring of the hydrolytic enzymes in lysosomes in real-time pathways would provide insights into the detailed role of lysosomal enzymes in the progression of diseases, and further aid in the development of early diagnosis and therapeutic strategies.^11–13^

Fluorescence imaging has revolutionized fundamental studies in life sciences by enabling the visualization, characterization, and measurement of biological processes at the molecular and cellular levels in living systems.^14–18^ In particular, activatable fluorescent probes in the near-infrared (NIR) window have shown a higher signal-to-noise ratio for *in vivo* imaging.^19–23^ Unfortunately, when it comes to the subcellular dimension, existing NIR activable probes are prone to dispersing throughout the cell, which largely compromises the fidelity of imaging for biomarkers within specific organelles.^24–27^ In the case of β-gal probes, for example, only a few lysosome-targetable fluorescent probes have been reported for β-gal thus far (Table 1).^28–32^ Most reported lysosome-targeting probes exhibited excitation and/or emission in the ultraviolet-visible region,^28–30^ which largely limits their further application *in vivo*. Recently, a lysosome-targeting probe Lyso-Gal exhibited desirable longer wavelength emission;^31^ however, the proximity of the lysosome-targeting moiety to the enzyme substrate raised concerns about steric hindrance toward the response. Moreover, anti-colocalization with mitochondria was not demonstrated with this probe. Alternatively, the organelle-targeting unit was grafted into the indolium moiety to afford the probe HCyXA-βGal, which exhibited light-up NIR fluorescence in ovarian cancer cells.^32^ Nevertheless, the positive charge properties of indolium brought about potential mitochondrial-targeting ability, leading to a bias in the fluorescence signal.^33,34^ In addition, the accessibility of small molecule-based lysosome-targeting probes in living objects remains to be further explored (Table 1), despite the success of nanodevices in mapping lysosomal enzyme activity *in vivo*.^35–37^ Given this, it is still challenging and highly desired to develop novel NIR lysosome-targeting probes for specific detection and visualization of biomarkers *in vivo*.

**Table 1:** Comparison of photophysical and kinetic properties of OELyso-Gal and other reported lysosome-targeting probes

Probe	*λ*_ex_/*λ*_em_ (nm)	*K*_m_ a (μM)	Response time^b^	LOD^c^ (U/mL)	Pearson's coefficient^d^	Imaging application	Ref.
FC-βgal	360/460; 460/560	—	30 min (150 U/L)	4.0 × 10^−5^	—	SKOV-3 cells	28
SRP	495/545	28.0	30 min (0.1 U)	4.19 × 10^−7^	—	Senescent vascular cells	29
BMZ-Gal	450/565	78.75	30 min (10 U)	3.6 × 10^−2^	0.71	SKOV-3 cells	30
Lyso-Gal	690/725	—	25 min (3 U/mL)	2.2 × 10^−2^	0.83	SKOV-3 cells	31
HCyXA-βgal	680/710	68.37	250 s (10 U/mL)	1.2 × 10^−2^	0.91	SKOV-3 cells and senescent WI-38 cells	32
OELyso-Gal	610/641	19.06	20 min (1.2 U/mL)	3.2 × 10^−3^	0.96	SKOV-3 tumor *in vivo*	This work

a *K*_m_, the enzyme kinetic parameter Michaelis–Menten constant, which represents the affinity of the probe to the enzyme.

b Response time, the time it takes for the probe to reach a plateau in fluorescence signal change (the concentration of the enzyme used).

c LOD, the limit of detection.

d The Pearson's co-localization coefficient between the lysosome-targeting probe and commercial lysosome tracker.

**Scheme 1:**
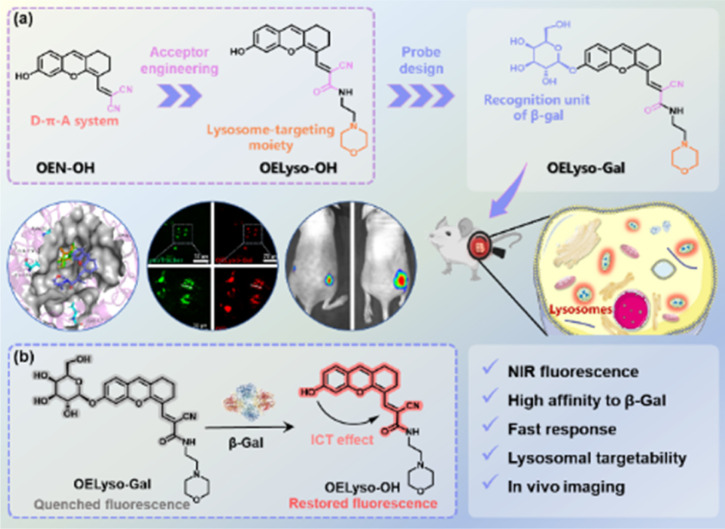
(a) Schematic illustration of acceptor engineering on the dihydroxanthene-based scaffold and rational design of the probe OELyso-Gal. (b) Proposed sensing mechanism of OELyso-Gal toward lysosomal β-gal.

Against this backdrop, we developed a general acceptor engineering strategy to construct NIR activatable probes with lysosome-targeting capability (Scheme 1). Upon isosteric replacement and additional functionalization, the electron-acceptor dicyanyl group in OEN-OH was replaced by cyanoacetamide with a morpholine ring to afford the fluorophore OELyso-OH, which maintained the NIR fluorescence but possessed additional lysosome-targeting ability. As a proof-of-concept, the enzymatic substrate β-galactopyranoside unit was directly grafted onto the fluorophore to afford the lysosome-targeting probe OELyso-Gal. By virtue of acceptor engineering, OELyso-Gal exhibited the advantages of high affinity (*K*_m_ = 19.06 μM) as well as fast response (20 min) to β-gal in the docking simulation and enzymatic kinetic investigations. In cell imaging, the fluorescence of OELyso-Gal could be activated specifically by lysosomal β-gal in SKOV-3 cells, hence allowing real-time imaging of intracellular β-gal with optimized fidelity. Moreover, the lysosome-targeting probe was found to be more sensitive to β-gal in cells and tissues, which boosted the dynamic monitoring of β-gal in ovarian tumour-bearing mice. Importantly, OELyso-Gal is the first lysosome-targeting NIR probe for tracking β-gal activity *in vivo*.

## Results and discussion

### Molecular design and synthesis

The dihydroxanthene-based scaffold OEN-OH, characterized as a donor–π–acceptor (D–π–A) system, has been utilized as a precursor for NIR probes with advantages of good photostability and excellent biocompatibility.^12,38^ Herein, the electron-acceptor dicyanyl group in OEN-OH was replaced by cyanoacetamide with a morpholine ring to afford the fluorophore OELyso-OH (Scheme 1). Considering the rule of isosteric replacement, the obtained OELyso-OH was expected to maintain the NIR fluorescence but possess additional lysosome-targeting ability. Moreover, the incorporation of a lysosome-targeting moiety may have minimal impact on the response toward the analyte of interest owing to the distant distribution. As proof of the concept, the enzymatic substrate β-galactopyranoside unit was directly grafted on the fluorophore to afford the lysosome-targeting probe OELyso-Gal. Upon substitution with the enzyme-trigger, the NIR fluorescence was expected to be quenched through hindering the intramolecular charge transfer (ICT) effect but intensive NIR fluorescence was observed after exposure toward β-gal. In living cells, the probe OELyso-Gal is expected to be preferentially accumulated in lysosomes and continuously activated by lysosomal β-gal, and thus may afford a significant advantage in terms of sensitivity and fidelity.

The dihydroxanthene-based scaffold OE-OH was prepared from a previous method,^39^ followed by Knoevenagel condensation with ethyl cyanoacetate to obtain the precursor OEE-OH. To introduce the organelle-targeting unit, the ester intermediately underwent an ammonolysis reaction with 4-(2-aminoethyl)morpholine to afford the lysosome-targeting fluorophore OELyso-OH, while the dicyanyl-substituted fluorophore OEN-OH was obtained from a Knoevenagel condensation between OE-OH and malononitrile.^40^ The two β-gal-activated probes OELyso-Gal and OEN-Gal were synthesized through a nucleophilic substitution reaction and deacetylation. The detailed synthetic routes are depicted in Schemes S1 and S2,† and the structures were characterized by ^1^H NMR, ^13^C NMR and HRMS.

### Spectroscopic investigation

**Fig. 1:**
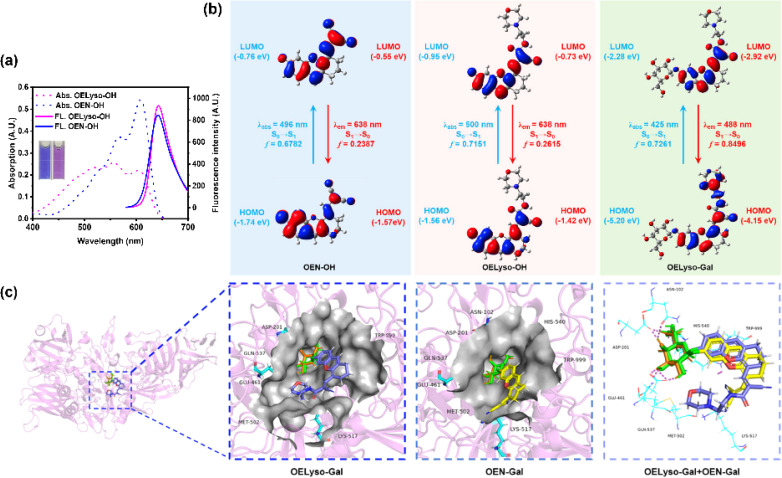
(a) The absorption and fluorescence spectra of OEN-OH (10 μM) and OELyso-OH (10 μM) in PBS (10 mM, pH = 7.4, containing 30% DMSO). Inset: the corresponding images of OEN-OH and OELyso-OH. (b) Calculated frontal molecular orbitals involved in the vertical excitation and emission of OEN-OH, OELyso-OH and OELyso-Gal. (c) Molecular docking simulation of the binding mode between OELyso-Gal (in blue)/OEN-Gal (in yellow) and β-gal (ligand of galactose, in orange).

Having obtained the fluorophores, the solvatochromic absorption and emission spectra of OELyso-OH and OEN-OH were recorded in different solvents. As shown in Fig. S13,† the two fluorophores exhibited a bathochromic shift in polar aprotic solvents with increasing orientational polarizabilities, and importantly, OELyso-OH exhibited better resistance toward the solvent polarity changes than OEN-OH. Such an observation was more evident in the absorption spectra in polar protic solvents (Fig. S14†) as well as the fluorescence spectra in different solvents (Fig. S15†). Of note, the advantage is also distinct in water, in which the fluorescence of OEN-OH was almost quenched while the one of OELyso-OH remained. To evaluate the efficiency of isosteric replacement, the optical properties of OELyso-OH and OEN-OH in PBS (10 mM, pH = 7.4, containing 30% DMSO) were investigated (Fig. 1a). Although the absorption spectra varied, both fluorophores exhibited a prominent emission peak at about 640 nm upon excitation at 560 nm, confirming that the fluorescence wavelength was not affected obviously by the introduction of acceptor engineering. A similar conclusion could also be obtained from the consistent wavelength in the solvatochromic investigation.

To further support the idea, density functional theory (DFT) and time-dependent density functional theory (TD-DFT) were implemented using Gaussian 09. As shown in Fig. 1b, both the highest occupied molecular orbital (HOMO) and lowest unoccupied molecular orbital (LUMO) in OELyso-OH and OEN-OH were located mainly in the whole fluorophore, and a similar energy gap between HOMO and LUMO could also be found in the two fluorophores, which could contribute to their similar intramolecular charge transfer (ICT) strength and thus similar optical behavior.^41^ Furthermore, the calculated vertical excitation and emission wavelengths of OELyso-OH and OEN-OH were very close, substantiating again the minimal effect of the isosteric replacement on the optical properties.^42^ By contrast, when a block unit was grafted on the hydroxyl group (*e.g.*, a β-galactopyranoside unit to afford OELyso-Gal), both the energy gap and the calculated wavelengths changed dramatically. The results manifest that the functionalized fluorophore OELyso-OH may provide a robust platform to develop activatable probes with distinct fluorescence changes as well as potential lysosomal targeting capability.

### Response mechanisms of OELyso-Gal

Inspired by the desired results, the behavior of the probe OELyso-Gal in response to β-gal was next characterized. First, the binding mode of OELyso-Gal to β-gal was investigated by molecular docking simulation. As shown in Fig. 1c and S16,† the recognition group β-galactopyranoside unit can enter the enzymatic pocket successfully and form hydrogen bonds with the surrounding amino acid residues, which demonstrates the high affinity of OELyso-Gal toward β-gal.^43,44^ More importantly, the receptor ends of the fluorophore were stuck outside the pocket as expected, suggesting that the introduction of morpholine rings may have little effect on the binding mode.^45,46^ In addition, the binding mode of OEN-Gal (marked in yellow) is highly overlapped with the one of OELyso-Gal (marked in blue). These results suggest that the acceptor engineering may have a modest effect on the affinity of probes to the enzyme, authenticating the accessibility of OELyso-Gal into the β-gal catalytic pocket for subsequent activation.

**Fig. 2:**
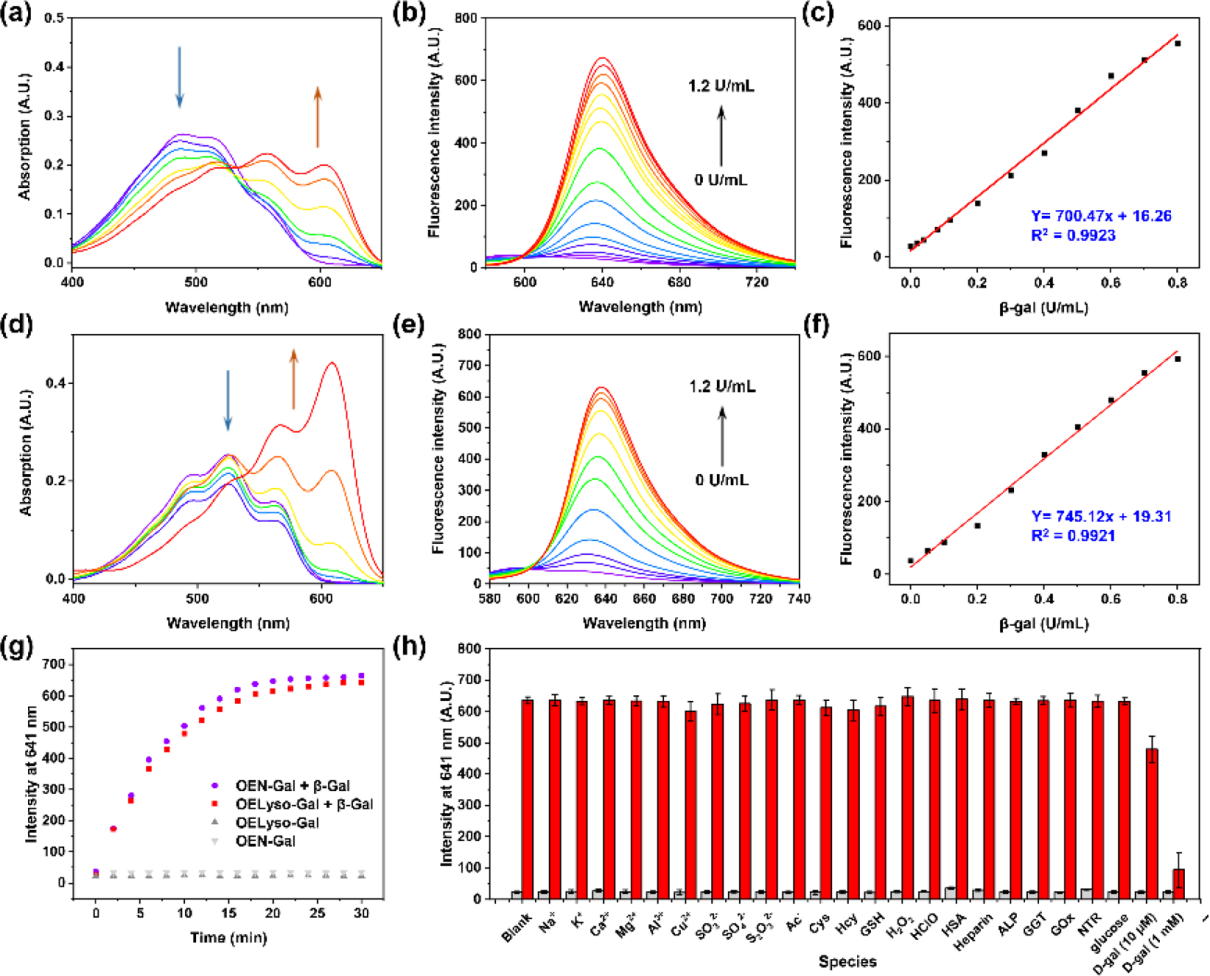
Spectral profiles of OELyso-Gal (10 μM) and OEN-Gal (10 μM) incubated with β-gal for 20 min. (a–c) Absorption spectra (a), fluorescence spectra (b) and fluorescence intensity (c) of OELyso-Gal incubated with β-gal. (d–f) Absorption spectra (d), fluorescence spectra (e) and fluorescence intensity (f) of OEN-Gal incubated with β-gal. (g) Time-dependent fluorescence intensity of OELyso-Gal (red blocks) and OEN-Gal (purple dots) in the presence of β-gal (1.2 U/mL). (h) Fluorescence intensity of OELyso-Gal at 641 nm upon incubation with β-gal in the absence or presence of various interferents.

To validate the enzymatic activation, the absorption and emission spectra of OELyso-Gal were inspected in the absence and presence of β-gal. Compared with the initial spectra, OELyso-Gal (10 μM) incubated with β-gal (1.2 U/mL) for 20 min exhibited a significant hypsochromic shift in absorption spectra (Fig. 2a), along with an intensive fluorescence enhancement in emission spectra (Fig. 2b). By contrast, the fluorescence enhancement could be attenuated by the competitive inhibitor d-galactose in a dose-dependent manner (Fig. S17†), proving that the optical changes were triggered specifically by the presence of β-gal. To verify the conclusion, high-performance liquid chromatography (HPLC) analysis was carried out. As shown in Fig. S18b,†OELyso-Gal itself exhibited a signal peak at about 5.9 min, but after incubation with β-gal, the peak decreased while another peak appeared at 6.9 min. As the incubation time prolonged, the newly emerged peak dominated in the whole elution, which was attributed to the fluorophore OELyso-OH (7.0 min). The further high-resolution mass spectrum (HRMS) analysis (Fig. S18c†) also indicated the hydrolysis of the β-galactoside bond in OELyso-Gal (*m/z*: calc., 570.2452; found, 570.2461) to release the fluorophore OELyso-OH (*m/z*: calc., 408.1923; found, 408.1928) after incubation with β-gal. These results supported the allowed enzymatic hydrolysis of the β-galactoside bond in OELyso-Gal by the catalysis of β-gal.

### Optical response of OELyso-Gal to β-gal

Having verified the enzymatic hydrolysis, the spectral response of OELyso-Gal to β-gal was characterized in detail. As shown in Fig. 2a, OELyso-Gal showed a red color with a broad absorption spectrum ranging from 400 to 600 nm. Upon incubation with β-gal (0–1.2 U/mL) in solution (PBS/DMSO = 7 : 3, V/V, pH 7.4) at 37 °C for 20 min, the absorption peak of OELyso-Gal at 525 nm decreased while two peaks emerged at around 567 and 609 nm. This also appeared as the solution color changed from red to violet. Of note, the color change and hypsochromic shift are consistent with the optical properties of the fluorophore OELyso-OH. Upon excitation at 560 nm, the fluorescence of the probe OELyso-Gal ranging from 580 to 740 nm increased with the concentration of β-gal in the range of 0–1.2 U/mL (Fig. 2b). A good linearity was found between the fluorescence intensity at 641 nm and β-gal concentration, which enables quantitative detection of β-gal ranging from 0 to 0.8 U/mL (Fig. 2c). The limit of detection (LOD) of OELyso-Gal was calculated to be 3.2 × 10^−3^ U/mL, a high value of sensitivity comparable with most of the probes. The optical titration experiment of OEN-Gal toward β-gal activities was also carried out. As depicted in Fig. 2d and e, a similar hypsochromic shift was observed in absorption spectra and a consistent fluorescence enhancement at 639 after incubation with β-gal (0–1.2 U/mL) for 20 min at 37 °C. The LOD of OEN-Gal was determined to be 3.0 × 10^−3^ U/mL (Fig. 2f).

After incubation with potentially interfering biological substances including cations, reduction anions, reactive oxygen species, amino acids, and enzymes with OELyso-Gal (10 μM) for 20 min, negligible fluorescence was observed, indicating that OELyso-Gal has excellent selectivity toward β-gal over other relevant interferents (Fig. 2h). Furthermore, the competitive experiment demonstrated that the presence of other interferents has a negligible effect on the fluorescence enhancement of OELyso-Gal except for the groups containing d-galactose (competitive inhibitor of β-gal). The pH effects also exhibited a desirable fluorescence response to β-gal in the pH range from 6.0 to 8.6 (Fig. S19†), which is also consistent with the good catalytic activity of β-gal in this range. These results demonstrate that OELyso-Gal exhibits the desired performance for sensitive detection of β-gal in biological matrices.

To further understand the enzymatic kinetics of probes, the fluorescence intensities of OELyso-Gal and OEN-Gal as a function of time were compared upon incubation with β-gal (1.2 U/mL). As depicted in Fig. 2g and S20,† the fluorescence increased quickly in 15 min, which reached a plateau at 20 min, indicative of the fast response of both probes to β-gal. Of note, the signal enhancement of OELyso-Gal (25.2 folds) was higher than that of OEN-Gal (18.3 folds). Furthermore, the Michaelis–Menten and Lineweaver–Burk equations were constructed.^40,45^ As shown in Fig. S21,† the kinetic parameter Michaelis constant (*K*_m_) of OELyso-Gal was calculated to be 19.06 μM, the lowest value reported for probes with lysosomal targetability (Table 1). The result indicates the favorable affinity of OELyso-Gal to β-gal, which could be attributed to the minimal steric hindrance of the probe to the enzymatic pocket by virtue of acceptor engineering, while the *K*_m_ of OEN-Gal was determined to be 16.24 μM. These results were consistent with the above molecular docking results (Fig. 1c). More importantly, OELyso-Gal with superior affinity to β-gal could be an ideal candidate for the fast detection and high-fidelity imaging of the enzyme in biological scenarios.

### Real-time fluorescence imaging of lysosomal β-gal in living cells

**Fig. 3:**
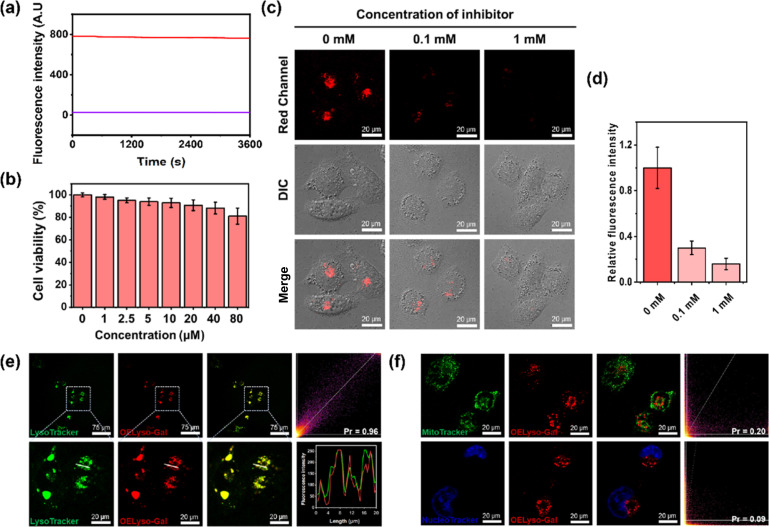
(a) The photostability of OELyso-Gal and OELyso-Gal incubated with β-gal. (b) Cell viability of SKOV-3 cells incubated with OELyso-Gal. (c and d) Fluorescence images and relative fluorescence intensity of OELyso-Gal (10 μM)-staining SKOV-3 cells treated with inhibitors (d-galactose, 100 μM or 1 mM). (e and f) Fluorescence imaging of SKOV-3 cells incubated with OELyso-Gal (10 μM) for 30 min and then stained with LysoTracker-Green (5 μM), MitoTracker-Green (1 μM), or Hoechst (1 μM) for another 30 min.

Inspired by the favorable performance of OELyso-Gal*in vitro*, its applicability for visualization of the endogenous β-gal in living cells was assessed. Before that, the cytotoxicity of OELyso-Gal against SKOV-3 cells was evaluated using MTT assay. No distinct effect was found on the cell viability (>90%) at concentrations less than 80 μM (Fig. 3b), indicating the excellent biocompatibility of OELyso-Gal. Another determinant requirement for the practical applications in long-term monitoring is the high photostability toward high-energy lasers.^38,47^ The photostability of OELyso-Gal was evaluated under the excitation of a 150 W xenon lamp. As shown in Fig. 3a, the signal intensities at 641 nm before and after β-gal incubation were relatively stable during the scanning period of 60 min, indicating the desirable resistance to photobleaching. Next, the probe was applied for tracking endogenous β-gal in SKOV-3 cells, a cell line of primary ovarian cancer with high expression of β-gal.^7,48^ As shown in Fig. S22,† red fluorescence was observed in SKOV-3 cells, in which the signals intensified with increasing concentration of the probe. Considering the fidelity of the signals, the working concentration of the probe is determined to be 10 μM. Later, to elucidate the specificity of the probe in cells, a competitive inhibition experiment was carried out in SKOV-3 cells. The red fluorescence weakened gradually with increasing concentration of d-galactose (Fig. 3c and d), substantiating that the fluorescence enhancement is highly specific to the activity of intracellular β-gal.

After that, to elucidate the subcellular organelle-targeting ability of OELyso-Gal, the co-localization imaging experiment was conducted. The SKOV-3 cells were incubated with OELyso-Gal for 30 min and then stained with the commercial tracker for lysosome (LysoTracker-Green), mitochondria (MitoTracker-Green), and nucleus (Hoechst 33342), respectively, for another 30 min. As shown in Fig. 3e, the red fluorescence produced by OELyso-Gal overlapped well with the green fluorescence rendered by LysoTracker Green. The fluorescence intensity profiles of OELyso-Gal and LysoTracker-Green in the regions of interest (ROI, given by a white line) were well-fitting, revealing the excellent lysosome-targeting ability of OELyso-Gal. Moreover, Pearson's co-localization coefficient between OELyso-Gal and LysoTracker-Green was calculated to be 0.96, a value higher than that of the reported lysosome-targeting probes for β-gal (Table 1). The result confirms the effectiveness of acceptor engineering in the targeting of organelles. By contrast, Pearson's co-localization coefficient between OELyso-Gal and MitoTracker-Green (0.20) or Hoechst 33342 (0.09) was much smaller (Fig. 3f). These results verify that the probe OELyso-Gal could be accumulated in the lysosomes and activated specifically by lysosomal β-gal in SKOV-3 cells.

**Fig. 4:**
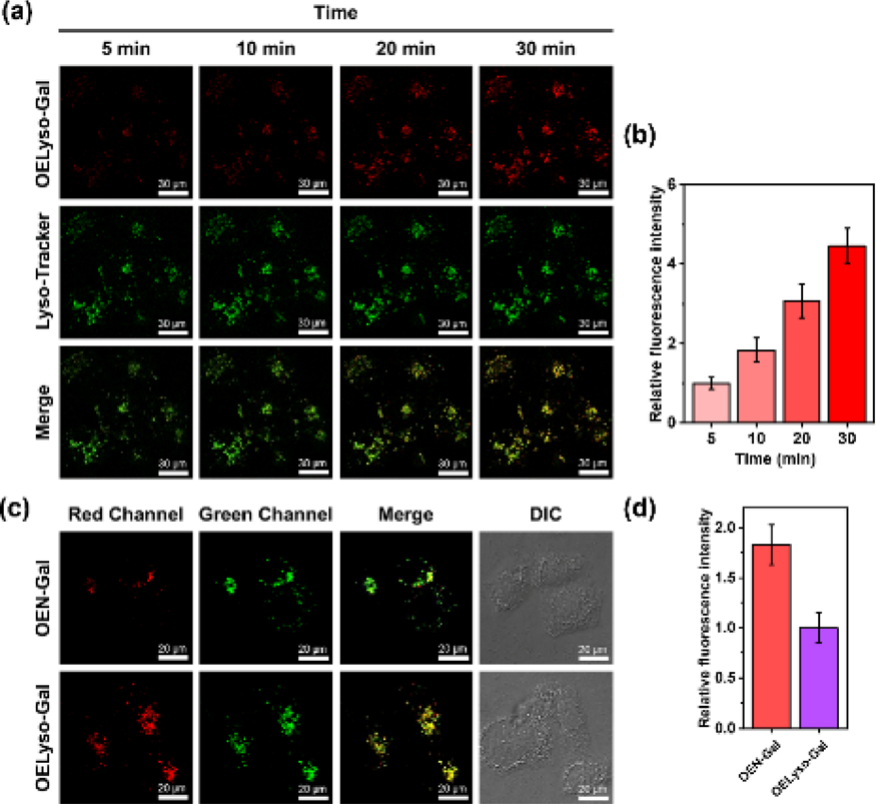
(a and b) Real-time fluorescence images and relative fluorescence intensity of OELyso-Gal (10 μM) in SKOV-3 cells pretreated with LysoTracker-Green (5 μM). Scale bar: 30 μm. (c and d) Fluorescence images and relative fluorescence intensity of LysoTracker-Green (5 μM)-staining SKOV-3 cells incubated with OEN-Gal (10 μM) or OELyso-Gal (10 μM), respectively. Scale bar: 20 μm.

To further substantiate the idea, the dynamic activation process of OELyso-Gal was illustrated in SKOV-3 cells pre-stained with LysoTracker-Green. As depicted in Fig. 4a and b, the fluorescence of OELyso-Gal exhibited persistent light-up at specific organelles. The red fluorescence was observed within 5 min and gradually intensified with prolonged incubation. More importantly, the time-lapsed red fluorescence derived from activated OELyso-Gal merged well with lysosomes labeled by LysoTracker-Green, which was consistent with the fact of lysosomal distribution of β-gal reported in previous research studies.^31,49^ The result demonstrates that the fluorescence of OELyso-Gal is indeed activated by lysosomal β-gal in ovarian cancer cells, allowing real-time fluorescence imaging of intracellular β-gal with optimal fidelity.

### Evaluation of β-gal activities in cells and tumour-bearing mice

Having demonstrated the success in specific imaging of lysosomal β-gal with OELyso-Gal, the significance of lysosomal targeting for β-gal detection in cells was revealed by comparing with the control probe OEN-Gal. Although no obvious difference was found in the optical response *in vitro*, a probe that could locate and accumulate in lysosomes is expected to provide higher signal intensities for lysosomal β-gal. As expected, upon the introduction of acceptor engineering, the fluorescence enhancement of OELyso-Gal was almost twice as large as that of OEN-Gal without lysosome-targeting ability (Fig. 4c and d). Similarly, their fluorescence signals overlapped well with those of LysoTracker-Green. The result suggests that the lysosome-targeting probe is more sensitive to imaging β-gal in cells, substantiating the role of the acceptor-engineering strategy proposed herein. Given its higher efficiency for endogenous β-gal detection, OELyso-Gal was utilized to evaluate the β-gal levels in different cells. OELyso-Gal was incubated with SKOV-3, HeLa, Hepa 1–6 and HepG2 cells for 30 min at 37 °C and then the fluorescence images were recorded. As shown in Fig. S24,† intensive fluorescence was found in SKOV-3 cells but faint fluorescence signals were observed in other cells, which have been found to have low β-gal levels.^19,40^ These results suggest that the probe OELyso-Gal could be an edge tool for discriminating β-gal-overexpressing ovarian cancer cells from other cells.

**Fig. 5:**
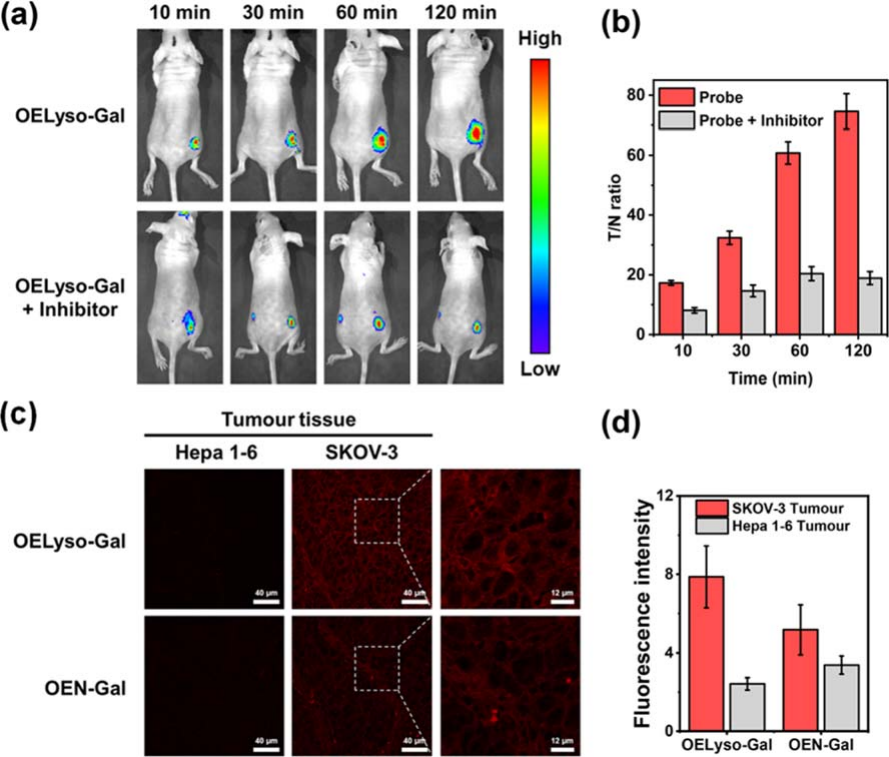
(a) Time-dependent fluorescence images of β-gal in the SKOV3 tumor-bearing mice intratumorally injected with OELyso-Gal (100 μM) or OELyso-Gal (100 μM) + d-galactose (1 mM). (b) Signal ratio between the tumor and normal site (*T*/*N*) in animals at different times. (c and d) Fluorescence images (c) and relative fluorescence intensity (d) of OELyso-Gal (10 μM)/OEN-Gal (10 μM) in Hepa 1–6 and SKOV-3 tumour tissue. Scale bar: 40 mm.

As a case in point, the practical feasibility of OELyso-Gal for real-time *in vivo* visualization of β-gal activity was evaluated in tumour-bearing mice. Before that, *in vitro* hemolysis assay was carried out. As shown in Fig. S25,† no significant hemolysis occurred at a concentration of OELyso-Gal up to 100 μM. Then, SKOV3 subcutaneous xenograft nude mice were treated with OELyso-Gal*via* intra-tumoural injection. As shown in Fig. 5a, distinct NIR fluorescence was collected specifically in the tumour region within 10 min, and gradually increased to the maximum within 120 min, indicating the quick activation of OELyso-Gal*in situ*. According to the intensity measurements in the region of interest, the signal ratio between the tumour and normal site (*T*/*N*) reached 74.6, indicating successful tumour identification (Fig. 5b). To further validate the finding that NIR signals were indeed activated by β-gal in ovarian tumours, the mice were preinjected with d-galactose for 30 min and then treated orthotopically with OELyso-Gal. The NIR fluorescence in the ovarian tumour attenuated a lot compared with that in the absence of inhibitors (*T*/*N* value decreased to 18.9), verifying that the increased fluorescence signals at the tumour site came from β-gal activated OELyso-Gal. Collectively, these results implied the potential of the probe OELyso-Gal as a promising tool for monitoring ovarian cancer *in vivo*.

Finally, to illustrate the early detection capability, we tested the accessibility of OELyso-Gal in tissue imaging. As shown in Fig. 5c, high-contrast fluorescence was observed in ovarian tumour slices while no fluorescence was observed in low β-gal-expressing liver tumour slices. The result demonstrates the capability of OELyso-Gal for discriminating between high and low β-gal-expressing tissues *in situ*. Moreover, the fluorescence intensity of OELyso-Gal in ovarian tumour tissue was found to be higher than that of OEN-Gal (Fig. 5d), which is consistent with the cell imaging in Fig. 4c. The result validates the observation that lysosomal specificity improves sensitivity in tissues and, more importantly, demonstrates the promise of OELyso-Gal in early detection in clinical scenarios.

## Conclusions

In summary, we successfully designed and constructed a lysosome-targeting dihydroxanthene-based NIR activatable probe, OELyso-Gal, for imaging disease-associated enzymes in lysosomes. OELyso-Gal exhibited high affinity and fast response to β-gal in the enzymatic kinetic investigations, revealing minimal steric hindrance effects from acceptor engineering. Notably, the *K*_m_ of OELyso-Gal was found to be 19.06 μM, the lowest value reported for probes with lysosomal targetability. Moreover, OELyso-Gal had a higher co-localization coefficient with the lysosome tracker than other reported probes, and such excellent lysosomal specificity was found to enhance the sensitivity of OELyso-Gal in living ovarian cancer cells and in an ovarian tumour section. Furthermore, the superiority of OELyso-Gal was demonstrated in real-time visualization of ovarian cancer in tumour-bearing mice, which is the first *in vivo* implementation with a lysosome-targeting probe activated by β-gal. We believe that this design strategy of acceptor engineering will provide inspirational insights into the development of customized fluorescent probes for monitoring disease-associated biomarkers within subcellular organelles.

## Data availability

Synthetic procedure, characterization, photophysical measurements, calculation results and fluorescence imaging can be found in the main text or the ESI.†

## Author contributions

B. Feng and F. Chu wrote and reviewed the manuscript. B. Feng synthesized the compounds. F. Chu performed biochemical experiments. Y. Fang performed the calculation experiments. M. Liu and X. Feng performed imaging experiment. J. Dong, and F. Chen reviewed and commented the manuscript. W. Zeng supervised the entire project.

## Conflicts of interest

The authors declare that they have no competing financial interests.

## Supplementary Material

SC-015-D4SC00487F-s001

## References

[cit1] Mahapatra K. K., Mishra S. R., Behera B. P., Patil S., Gewirtz D. A., Bhutia S. K. (2021). Cell. Mol. Life Sci..

[cit2] Gao Y., Hu Y., Liu Q., Li X., Li X., Kim C. Y., James T. D., Li J., Chen X., Guo Y. (2021). Angew Chem. Int. Ed. Engl..

[cit3] Boustany R.-M. N. (2013). Nat. Rev. Neurol..

[cit4] Edward J. G., Adam B., Janice B. S., Erin L. A., Ronald C. P., Bruce L. M., Dimitrios K. (2015). Neurology.

[cit5] Cai Y., Zhou H., Zhu Y., Sun Q., Ji Y., Xue A., Wang Y., Chen W., Yu X., Wang L., Chen H., Li C., Luo T., Deng H. (2020). Cell Res..

[cit6] Chatterjee S. K., Bhattacharya M., Barlow J. J. (1979). Cancer Res..

[cit7] Asanuma D., Sakabe M., Kamiya M., Yamamoto K., Hiratake J., Ogawa M., Kosaka N., Choyke P. L., Nagano T., Kobayashi H., Urano Y. (2015). Nat. Commun..

[cit8] Chai X., Han H. H., Sedgwick A. C., Li N., Zang Y., James T. D., Zhang J., Hu X. L., Yu Y., Li Y., Wang Y., Li J., He X. P., Tian H. (2020). J. Am. Chem. Soc..

[cit9] Yao Y., Zhang Y., Yan C., Zhu W. H., Guo Z. (2021). Chem. Sci..

[cit10] Chen J. A., Pan H., Wang Z., Gao J., Tan J., Ouyang Z., Guo W., Gu X. (2020). Chem. Commun..

[cit11] Trinh N., Jolliffe K. A., New E. J. (2020). Angew Chem. Int. Ed. Engl..

[cit12] Feng B., Chu F., Bi A., Huang X., Fang Y., Liu M., Chen F., Li Y., Zeng W. (2023). Biotechnol. Adv..

[cit13] Xiong J., Cheung Y. K., Fong W. P., Wong C. T. T., Ng D. K. P. (2023). Chem. Commun..

[cit14] Crosby D., Bhatia S., Brindle K. M., Coussens L. M., Dive C., Emberton M., Esener S., Fitzgerald R. C., Gambhir S. S., Kuhn P., Rebbeck T. R., Balasubramanian S. (2022). Science.

[cit15] Rowe S. P., Pomper M. G. (2022). CA-Cancer J. Clin..

[cit16] Hu Z. H., Fang C., Li B., Zhang Z. Y., Cao C. G., Cai M. S., Su S., Sun X. W., Shi X. J., Li C., Zhou T. J., Zhang Y. X., Chi C. W., He P., Xia X. M., Chen Y., Gambhir S. S., Cheng Z., Tian J. (2020). Nat. Biomed. Eng..

[cit17] Goshisht M. K., Tripathi N., Patra G. K., Chaskar M. (2023). Chem. Sci..

[cit18] Zhang Y. M., Zhang Z. X., Wu M. M., Zhang R. (2023). ACS Meas. Sci. Au.

[cit19] Feng B., Chu F., Huang X., Fang Y., Liu M., Liu M., Chen F., Zeng W. (2023). Sens. Actuators, B.

[cit20] Lou H., Ji A., Qu C., Duan S., Liu H., Chen H., Cheng Z. (2022). Chem. Eng. J..

[cit21] Yao S. K., Chen Y. C., Ding W. Z., Xu F. W., Liu Z. P., Li Y. H., Wu Y. P., Li S. M., He W. J., Guo Z. J. (2023). Chem. Sci..

[cit22] Gu K., Xu Y., Li H., Guo Z., Zhu S., Zhu S., Shi P., James T. D., Tian H., Zhu W. H. (2016). J. Am. Chem. Soc..

[cit23] Shi L., Yan C., Ma Y., Wang T., Guo Z., Zhu W. H. (2019). Chem. Commun..

[cit24] Wu X., Wang R., Kwon N., Ma H., Yoon J. (2022). Chem. Soc. Rev..

[cit25] Liu J., Ma X., Cui C., Chen Z., Wang Y., Deenik P. R., Cui L. (2021). J. Med. Chem..

[cit26] Gu K., Qiu W., Guo Z., Yan C., Zhu S., Yao D., Shi P., Tian H., Zhu W. H. (2019). Chem. Sci..

[cit27] Yu Q., Zhang L., Jiang M., Xiao L., Xiang Y., Wang R., Liu Z., Zhou R., Yang M., Li C., Liu M., Zhou X., Chen S. (2023). Angew Chem. Int. Ed. Engl..

[cit28] Huang J., Li N., Wang Q., Gu Y., Wang P. (2017). Sens. Actuators, B.

[cit29] Kim E.-J., Podder A., Maiti M., Lee J. M., Chung B. G., Bhuniya S. (2018). Sens. Actuators, B.

[cit30] Li Y., Ning L., Yuan F., Zhang T., Zhang J., Xu Z., Yang X. F. (2020). Anal. Chem..

[cit31] Li X., Pan Y., Chen H., Duan Y., Zhou S., Wu W., Wang S., Liu B. (2020). Anal. Chem..

[cit32] Pan H., Chai X., Zhang J. (2023). Chin. Chem. Lett..

[cit33] Fan L., Zan Q., Wang X., Yu X., Wang S., Zhang Y., Yang Q., Lu W., Shuang S., Dong C. (2022). Chem. Eng. J..

[cit34] Huang Y., Zhang Y., Huo F., Chao J., Yin C. (2022). Chem. Eng. J..

[cit35] Dan K., Veetil A. T., Chakraborty K., Krishnan Y. (2019). Nat. Nanotechnol..

[cit36] Zhang H. Y., Wu M. M., Ta H. T., Xu Z. P., Zhang R. (2023). Adv. Mater. Technol..

[cit37] Fan Y., Li F., Chen D. (2014). Biomaterials.

[cit38] Feng B., Ma Y., Zheng F., Huang X., Feng X., Zhang K., Liu L., Chen F., Zeng W. (2023). Chem. Eng. J..

[cit39] Zhou D. Y., Li Y., Jiang W. L., Tian Y., Fei J., Li C. Y. (2018). Chem. Commun..

[cit40] Pang X., Li Y., Zhou Z., Lu Q., Xie R., Wu C., Zhang Y., Li H. (2020). Talanta.

[cit41] Hou F., Liu X., Hao X., Li G., Lu F., Deng Q. (2021). Dyes Pigm..

[cit42] Tang Z., Zhou P. (2020). J. Phys. Chem. B.

[cit43] Li Z., Cheng J., Huang L., Li W., Zhao Y., Lin W. (2021). Anal. Chem..

[cit44] Chen S., Niu K., Wang L., Wu Y., Hou S., Ma X. (2022). Anal. Chim. Acta.

[cit45] Chen S., Wang L., Ma X., Wu Y., Hou S. (2022). Sens. Actuators, B.

[cit46] Yang L., Liu G., Chen Q., Wan Y., Liu Z., Zhang J., Huang C., Xu Z., Li S., Lee C. S., Zhang L., Sun H. (2022). Anal. Chem..

[cit47] Feng B., Zhu Y., Wu J., Huang X., Song R., Huang L., Feng X., Zeng W. (2021). Chin. Chem. Lett..

[cit48] Huang J., Jiang Y., Li J., Huang J., Pu K. (2021). Angew Chem. Int. Ed. Engl..

[cit49] Lee B. Y., Han J. A., Im J. S., Morrone A., Johung K., Goodwin E. C., Kleijer W. J., DiMaio D., Hwang E. S. (2006). Aging Cell.

