# Supplementary material for: High-fidelity imaging of a tumour-associated lysosomal enzyme with an acceptor engineering-boosted near-infrared fluorescent probe
Source: Chem Sci. 2024 Apr 16;15(19):7324–31. doi: 10.1039/d4sc00487f (PMC11095509; doi:10.1039/d4sc00487f)
Supplement: SC-015-D4SC00487F-s001 [file SC-015-D4SC00487F-s001.pdf]

Electronic Supplementary Material (ESI) for Chemical Science.
This journal is © The Royal Society of Chemistry 2024

Supporting information for

High-fidelity imaging of tumour-associated lysosomal enzyme with an acceptor engineering-boosted near-infrared fluorescent probe

Bin Feng [a,b], Feiyi Chu [a,b], Yanpeng Fang [a,b], Min Liu [a,c], Xueping Feng [c], Jie Dong [a,b], Fei Chen [a,b], and Wenbin Zeng [*a,b]

[a] Xiangya School of Pharmaceutical Sciences, Central South University, Changsha, 410013, PR China.

[b] Hunan Key Laboratory of Diagnostic and Therapeutic Drug Research for Chronic Diseases, Changsha, China

[c] Xiangya Hospital, Central South University, Changsha, 410013, PR China.

*Corresponding author. Tel/Fax: 0086-731-82650459. E-mail address: wbzeng@hotmail.com.

Experimental Section

Materials and instruments

All chemical reagents were obtained from commercial sources and used without further refinement. *β*-galactosidase (*E. coli. β*-gal, 5kU) was obtained from Sigma-Aldrich Co., Ltd (Shanghai, China). All solutions and buffers were prepared using deionized water that had been passed through a water ultra-purification system. ^{1}H-NMR spectra and ^{13}C-NMR spectra were recorded on a Bruker AV-500 spectrometer (Rheinstetten, Germany). High-resolution mass spectra (HRMS) were measured on a Thermo Scientific instrument. The pH measurements were conducted with Rex PHS-3C pH meter. The sizes of formed particles were determined via a Zestier Nano ZS (Malvern Instruments Ltd., U.K.). High-performance liquid chromatography (HPLC) analysis was performed on an Agilent 1100 system with a C8 column (American). A UV-2450 UV-visible spectrophotometer (Shimadzu, Japan) was used to measure the UV-vis spectra. A Hitachi F-2700 fluorescence spectrophotometer (Hitachi, Japan) was employed to record the fluorescence spectra with slits of 5 nm excitation and 5 nm emission. All these processes were implemented at 25 °C using standard quartz cuvettes with a 10-mm light path. A Leica TCS SP8 (MP+X) confocal laser scanning microscope (Leica, Germany) was employed to collect the fluorescent cell images.

Synthesis of OELyso-Gal

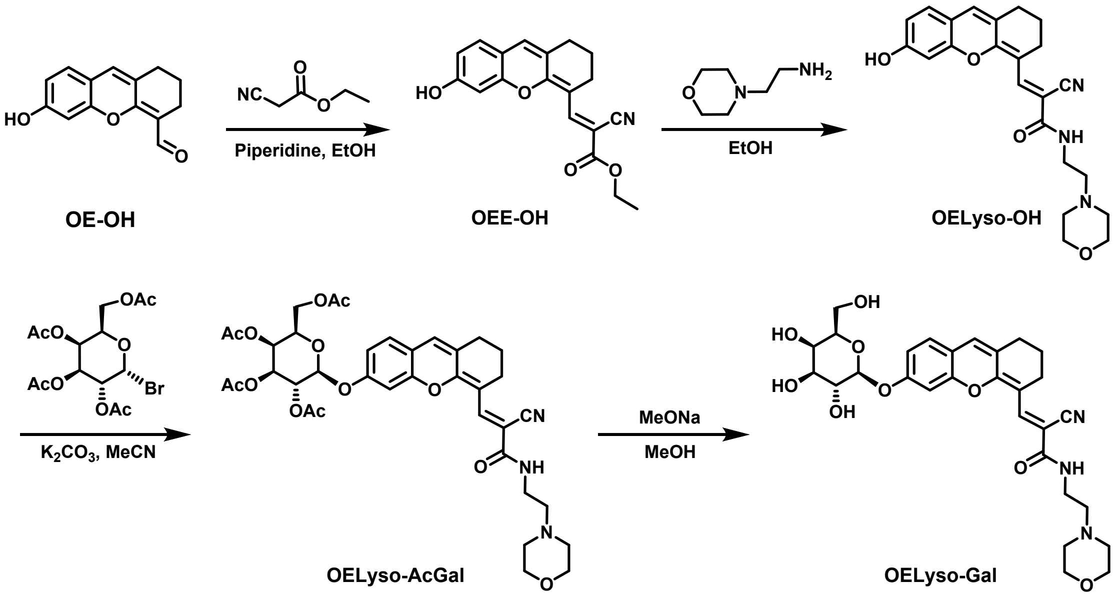

Scheme S1. Synthesis of probe **OELyso-Gal**.

Synthesis of **OEE-OH**. To ethyl cyanoacetate (339 mg, 3 mmol), piperidine (17 mg, 0.2 mmol)

in ethanol (10 mL) was added compound **OE-OH** (456 mg, 2 mmol) and the solution was refluxed at 80 °C for 3 h. The reaction mixture was filtered and the precipitate was washed with ice ethanol (20 mL x 2) and dried overnight to afford compound **OEE-OH** as a brown solid (600 mg, 93%). ^{1}H NMR (400 MHz, DMSO-*d₆*) δ 10.41 (s, 1H), 8.35 (s, 1H), 7.21 (dd, *J* = 8.4, 1.9 Hz, 1H), 6.99 (s, 1H), 6.68 – 6.62 (dd, *J* = 8.4, 1.9 Hz, 1H), 6.57 (s, 1H), 4.22 (q, *J* = 7.1 Hz, 2H), 2.73 (t, *J* = 5.5 Hz, 2H), 2.54 – 2.49 (m, 2H), 1.80 – 1.65 (m, 2H), 1.29 (t, *J* = 7.1 Hz, 3H). ^{13}C NMR (100 MHz, DMSO-d_6) δ 164.76, 160.86, 158.78, 153.84, 146.34, 130.74, 128.91, 125.32, 118.36, 113.89, 113.49, 108.26, 101.93, 91.24, 61.59, 28.75, 25.16, 20.62, 14.61. HR-MS (m/z): calculated for $C_{19}H_{18}NO_4^-$ $[M-H]^-$, 322.1085; found, 322.1008.

Synthesis of **OELyso-OH**. To 4-(2-aminoethyl) morpholine (260 mg, 2 mmol) in ethanol (5 mL) was added compound **OEE-OH** (485 mg, 1.5 mmol) and the solution was refluxed at 120 °C under nitrogen atmosphere for 8 h. The resulting mixture was concentrated in vacuo, washed with water (50 mL) and extracted with EtOAc (50 mL). The combined organic layers were dried over anhydrous $MgSO_4$, filtered and concentrated in vacuo. The residue was purified by column chromatography to give a red solid (381 mg, 62%). ^{1}H NMR (500 MHz, DMSO-d_6) δ 10.32 (s, 1H), 8.36 (s, 1H), 7.76 (s, 1H), 7.20 (d, *J* = 8.4 Hz, 1H), 6.93 (s, 1H), 6.62 (dd, *J* = 8.4, 2.2 Hz, 1H), 6.58 (d, *J* = 2.2 Hz, 1H), 3.61 – 3.55 (m, 4H), 3.32 (d, *J* = 6.1 Hz, 2H), 2.75 (t, *J* = 5.8 Hz, 2H), 2.59 – 2.52 (m, 2H), 2.44 (t, *J* = 6.1 Hz, 2H), 2.41 (s, 4H), 1.79 – 1.68 (m, 2H). ^{13}C NMR (125 MHz, DMSO-d_6) δ 162.87, 160.40, 156.79, 153.83, 143.86, 128.62, 128.58, 125.65, 119.29, 113.81, 112.90, 107.95, 102.04, 96.08, 57.41, 55.35, 53.64, 37.21, 28.93, 25.61, 20.86. HR-MS (m/z): calculated for $C_{23}H_{24}N_3O_4^-$ $[M-H]^-$, 406.1767; found, 406.1787.

Synthesis of **OELyso-AcGal.** A solution of acetonitrile (5 mL) was added with K_2CO_3 (207 mg, 1.5 mmol) and **OELyso-OH** (204 mg, 0.5 mmol). Tetra-O-acetyl-α-D-galactopyranosyl-1-bromide (393 mg, 0.9 mmol) in acetonitrile (1 mL) was added slowly and the mixture was stirred at 25 °C for 24 h. The reaction mixture was filtered under vacuum, washed with water (25 mL) and extracted with EtOAc (25 mL x 2). The combined organic layers were dried over anhydrous $MgSO_4$, filtered and concentrated in vacuo. The residue was purified by column chromatography to afford a red solid (218 mg, 59%). 1H NMR (500 MHz, DMSO-d6) δ 8.40 (s, 1H), 7.81 (t, J = 5.4 Hz, 1H), 7.34 (d, J = 8.5 Hz, 1H), 6.97 (s, 1H), 6.86 (d, J = 1.8 Hz, 1H), 6.78 (dd, J = 8.5, 2.1 Hz, 1H), 5.65 (d, J = 7.2 Hz, 1H), 5.38 (s, 1H), 5.24 (dd, J = 11.2, 3.9 Hz, 2H), 4.52 (t, J = 6.1 Hz, 1H), 4.19 (s, 1H),

4.12 (s, 1H), 3.59 – 3.56 (m, 4H), 3.34 – 3.30 (m, 2H), 2.77 (d, J = 5.5 Hz, 2H), (d, J = 6.7 Hz, 2H), 2.41 (s, 4H), 2.16 (s, 3H), 2.05 (s, 3H), 2.01 (s, 3H), 1.96 (s, 3H), 1.75 2.56 (d, J = 5.3 Hz, 2H), 2.45 (dd, J = 6.3, 3.2 Hz, 2H). 13C NMR (126 MHz, DMSO-d6) δ 170.43, 170.41, 169.98, 169.67, 162.47, 158.16, 156.11, 153.42, 144.01, 128.55, 127.92, 127.44, 118.99, 116.82, 113.49, 108.85, 102.91, 97.54, 97.46, 71.20, 70.56, 68.63, 67.75, 67.47, 67.39, 57.38, 53.62, 37.23, 29.04, 25.58, 20.89, 20.84, 20.79, 20.76, 20.71. HR-MS (m/z): calculated for $C_{37}H_{44}N_3O_{13}^+$ $[M+H]^+$, 738.2874; found, 738.2891.

Synthesis of **OELyso-Gal.** To a solution of compound **OELyso-AcGal** (148 mg, 0.2 mmol) in methanol (5 mL) was added with sodium methoxide (11 mg, 0.2 mmol) in methanol (4 mL) and the mixture was stirred at 25 °C overnight. After that, the solution was neutralized with acetic acid (0.1 mL) and the solvents were removed under reduced pressure. The residue was purified by column chromatography to afford a red solid (47 mg, 41%). ^{1}H NMR (500 MHz, DMSO-d_6) δ 8.37 (s, 1H), 7.90 (s, 1H), 7.29 (d, J = 8.6 Hz, 1H), 6.96 (s, 1H), 6.92 (d, J = 2.2 Hz, 1H), 6.84 (dd, J = 8.6, 2.2 Hz, 1H), 5.24 (d, J = 4.9 Hz, 1H), 4.96 (d, J = 7.6 Hz, 1H), 4.93 (d, J = 5.2 Hz, 1H), 4.75 (s, 1H), 4.60 (d, J = 4.5 Hz, 1H), 3.73 (t, J = 3.5 Hz, 1H), 3.66 (t, J = 6.2 Hz, 1H), 3.58 (m, 4H), 3.50 (s, 4H), 3.39 – 3.30 (m, 4H), 2.76 (t, J = 5.7 Hz, 2H), 2.58 – 2.54 (m, 2H), 2.47 (s, 4H), 1.80 – 1.66 (m, 2H). ^{13}C NMR (125 MHz, DMSO-d_6) δ 162.88, 159.65, 156.37, 153.44, 143.90, 128.32, 127.86, 127.28, 119.10, 116.02, 113.57, 108.54, 103.24, 101.33, 97.14, 75.96, 73.59, 70.76, 68.42, 66.47, 60.67, 57.27, 53.48, 31.99, 29.00, 159.65, 156.37, 153.44, 143.90, 128.32, 127.86, 127.28, 119.10, 116.02, 113.57, 108.54, 103.24, 101.33, 97.14, 75.96, 73.59, 70.76, 68.42, 66.47, 60.67, 57.27, 53.48, 31.99, 29.00, 25.70, 20.77. HR-MS (m/z): calculated for $C_{29}H_{36}N_3O_9^+$ $[M+H]^+$, 570.2452; found, 570.24615.

Synthesis of OEN-Gal

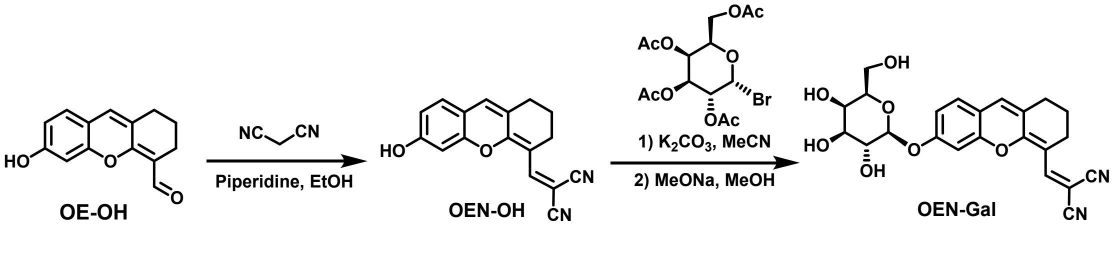

Scheme S2. Synthesis of probe **OEN-Gal**.

Synthesis of **OEN-OH**. To malononitrile (99 mg, 1.5 mmol), piperidine (17 mg, 0.1 mmol) in

ethanol (10 mL) was added compound **OE-OH** (228 mg, 1 mmol) and the solution was refluxed at 80 °C for 1 h. The reaction mixture was filtered and the precipitate was washed with ice ethanol (20 mL x 2) and dried overnight to afford compound **OEN-OH** as a red solid (260 mg, 94%). ^{1}H NMR (500 MHz, DMSO-d_6) δ 10.58 (s, 1H), 8.04 (s, 1H), 7.32 (d, J = 8.5 Hz, 1H), 7.26 (s, 1H), 6.85 (d, J = 2.1 Hz, 1H), 6.73 (dd, J = 8.5, 2.1 Hz, 1H), 2.69 (t, J = 6.0 Hz, 2H), 2.60 – 2.53 (t, J = 11.9 Hz, 3H), 1.72 (dt, J = 11.9, 6.0 Hz, 3H). ^{13}C NMR (125 MHz, DMSO-d_6) δ 161.42, 159.75, 154.21, 150.01, 133.24, 129.15, 124.97, 118.31, 116.86, 114.22, 114.06, 109.32, 102.63, 66.75, 28.48, 24.74, 20.50.

Synthesis of **OEN-Gal**. To a solution of acetonitrile (10 mL) of compound K_2CO_3 (207 mg, 1.5 mmol) and **OEN-OH** (138 mg, 0.5 mmol) was added with tetra-O-acetyl-*α-D*-galactopyranosyl-1-bromide (393 mg, 0.9 mmol) in acetonitrile (1 mL) slowly and the mixture was stirred at 25 °C for 24 h. The reaction mixture was filtered under vacuum, washed with water (25 mL) and extracted with EtOAc (25 mL x 2). The combined organic layers were dried over anhydrous $MgSO_4$, filtered and concentrated in vacuo. The residue was dissolved in methanol (5 mL), and added slowly sodium methoxide (22 mg, 0.4 mmol) in methanol (4 mL) was added slowly. The mixture was stirred at 25 °C overnight. After that, the solution was neutralized with acetic acid (0.2 mL) and the solvents were removed under reduced pressure. The residue was purified by column chromatography to afford a red solid (38 mg, 17.4%) ^{1}H NMR (600 MHz, DMSO-d_6) δ 8.16 (s, 1H), 7.44 (d, J = 8.5 Hz, 1H), 7.35 (s, 1H), 7.24 (s, 1H), 6.95 (d, J = 8.5 Hz, 1H), 5.25 (s, 1H), 4.99 (s, 1H), 4.92 (s, 1H), 4.69 (s, 1H), 4.57 (s, 1H), 3.70 (m, 2H), 3.56 (m, 2H), 3.49 (s, 1H), 3.42 (s, 1H), 2.75 (s, 2H), 2.62 (s, 2H), 1.76 (s, 2H). ^{13}C NMR (150 MHz, DMSO-d_6) δ 160.35, 159.52, 153.76, 150.45, 132.44, 128.87, 126.72, 118.10, 116.63, 116.02, 114.70, 109.88, 103.52, 101.00, 76.05, 73.72, 70.61, 68.43, 68.03, 60.64, 28.57, 24.79, 20.43.

Investigation to solvent effect of OELyso-OH and OEN-OH.

OELyso-OH/OEN-OH was dissolved in DMSO to obtain the stock solution (2 mM), and then diluted with different solvents to get samples containing **OELyso-OH/OEN-OH** (10 μM). Then, the UV-vis spectra and fluorescence spectra were recorded. To investigate the photostability, **OELyso-OH/OEN-OH** (10 μM) was prepared in DMSO and the fluorescence spectra were recorded under the excitation of a 150 W xenon lamp for different durations.

Methods of theoretical calculations

Calculations of optimal geometries and electronic structures of **OEN-OH**, **OELyso-OH** and **OELyso-Gal** were carried out by density functional theory (DFT) and time-dependent density functional theory (TDDFT) at the B3LYP/6-31G(d,p) level [1]. The starting structures with different conformational isomers were considered to ensure that the optimized structure corresponds to a global minimum. All these calculations were carried out with Gaussian 09W software (vacuum was employed in all the calculations). The obtained data were analyzed by GaussView 5.0 software.

Method of molecular docking simulation

Molecular docking simulations were carried out on MOE2019 software. The crystal structure of *E. coli β*-gal (PDB ID: 1JYN) was downloaded from RCSB and underwent a series of optimizations (completing the protein sequence, deleting repeated subunits, adding hydrogen atoms, removing all water molecules and ligands). Different conformations of **OELyso-OH/OEN-OH** were searched (n=10000) and the docking cycles and the parameters between ligand and protein were set according to the defaults.

HPLC traces for response behavior

The probe **OELyso-Gal** was incubated with *β*-gal for different durations (5 min and 20 min) at 37°C and then the incubation system was monitored by high-performance liquid chromatography (HPLC) on an Agilent Technologies 1100 Infinity LC system. While, **OELyso-Gal** and fluorophore **OELyso-OH** were used as the reference. The mobile phases were degassed with an ultrasonic apparatus for 10 min. Mobile phase: A: water, B: acetonitrile; elution: 0 - 3 min 50% B (isocratic), 3 - 7 min 50 - 80% B (gradient), 7 - 8 min 80% B (isocratic). Injection volume: 10 µL; flow rate: 1.0 mL/min; detection wavelength: 365 nm.

Kinetic assay

Various concentrations of **OELyso-Gal/OEN-Gal** (1, 2, 4, 6, 8, 10, 15, 20, 30, 40 µM) were incubated with *β*-gal (1.2 U/mL) at 37 °C for 5 min in PBS containing 30% (v/v) DMSO. After incubation, the absorption spectra of the mixture at 610 nm were measured for quantification analyses. The initial reaction velocity (µM min^{-1}) was calculated, plotted against the concentration of **OELyso-Gal/OEN-Gal**, and fitted to a Michaelis-Menten curve. The kinetic parameters were calculated according to the Michaelis-Menten equation shown below:

$$V = V\_max \ ([S])/(K\_m + [S])$$

where V is the initial velocity, [S] is the substrate concentration, Km is the Michaelis constant, and V_{max} is the maximum initial reaction rate.

Cytotoxicity assay against SKOV-3 cells

Human SKOV-3 cells were cultured in Roswell Park Memorial Institute (RPMI) 1640 medium supplemented with 10% fetal bovine serum (FBS), 100 mg/ml penicillin and 100 μg/ml streptomycin in a humidified incubator containing 5% CO_2 at 37 °C. Then the cytotoxic effect of probe **OELyso-Gal** (0, 1, 2.5, 5, 10, 20, 40, 80 μM, containing 0.5% DMSO in medium) was evaluated in SKOV-3 cells using the standard CCK8 assay.

Fluorescence imaging of SKOV-3 cells

For imaging experiments, SKOV-3 cells were seeded on confocal dishes and allowed to adhere at 37℃ and 5% CO_2 for 12 h. After that, the adherent cells were incubated with medium containing probe **OELyso-Gal** at concentrations of 0, 5 μM, 10 μM, and 20 μM for 30 min at 37℃, respectively. Then the cells were washed three times with PBS buffer, and imaged in medium under the confocal laser scanning microscope (CLSM). The NIR channel signals of **OELyso-Gal** were collected at 620-680 nm (λ_{ex} = 594 nm).

To investigate the effect of inhibitor, the adherent cells were co-incubated with/without different concentrations of *D*-galactose (0.1 or 1 mM) prepared in medium for 30 min at 37 °C in an incubator with 5% CO_2. Then, the living cells were incubated with **OELyso-Gal** (10 μM, containing 0.5% DMSO in medium) for another 30 min at 37 °C. After washing the cells with PBS three times, the confocal fluorescence images were acquired at NIR channels (620-680 nm) upon excitation at 594 nm under CLSM.

Fluorescence colocalization assay

After being adhered in confocal dishes for 12 h, SKOV-3 cells were incubated with **OELyso-Gal** (10 μM, containing 0.5% DMSO in medium) for 30 min and then stained with LysoTracker-Green (5 μM), MitoTracker-Green (1 μM), or Hoechst (1 μM) for another 30 min at 37 °C, respectively. After washing the cells three times with PBS, the confocal fluorescence images were acquired at NIR channels (620-680 nm) upon excitation at 594 nm, green channels (490–530 nm) upon excitation at 458 nm for LysoTracker-Green, Green channels (490–530 nm) upon excitation at 488 nm for MitoTracker-Green, and blue channels (430–480 nm) upon excitation at 405 nm for Hoechst.

To investigate applicability in the real-time fluorescence imaging, the adhered cells were

pretreated with LysoTracker-Green (5 μM) for 30 min and then incubated with **OELyso-Gal** (10 μM, containing 0.5% DMSO in medium). The confocal fluorescence images were collected at 5 min, 10 min, 20 min, and 30 min, respectively. For comparison with **OEN-Gal**, the LysoTracker-Green (5 μM)-pretreated cells were stained with **OELyso-Gal** (10 μM, containing 0.5% DMSO in medium) and **OEN-Gal** (10 μM, containing 0.5% DMSO in medium) for 30 min at 37 °C, respectively. After that, the signals of **OELyso-Gal/ OEN-Gal** were acquired at NIR channels (620-680 nm) upon excitation at 594 nm while the one of LysoTracker-Green was at green channels (490–530 nm) upon excitation at 458 nm.

Fluorescence imaging in different cells

HeLa, Hepa 1-6, and HepG2 cells were cultured in Dulbecco's modified Eagle medium (DMEM) supplemented with 10% fetal bovine serum (FBS), 100 μg/mL penicillin and 100 μg/mL streptomycin in a humidified incubator containing 5% CO_2 at 37 °C. After being adhered in confocal dishes for 12 h, SKOV-3, HeLa, Hepa 1-6 and HepG2 cells were incubated with medium containing probe **OELyso-Gal** (10 μM, containing 0.5% DMSO in medium) for another 30 min at 37 °C, respectively. After washing the cells with PBS three times, the confocal fluorescence images were acquired at NIR channels (620-680 nm) upon excitation at 594 nm.

Hemolysis assay

Healthy mice blood (2 mL) was donated from female mice. RBCs were collected by centrifugation at 1500 rpm for 5 min, washed with normal saline (0.9% NaCl) until the supernatant was clear, and resuspended using normal saline (50 mL) to prepare 4% erythrocyte solution. Then, different concentrations (1, 2, 5, 10, 20, 40, 60, 80, 100 μM) of **OELyso-Gal** dissolved in normal saline solutions were added to the same-volume 4% erythrocyte solution in centrifuge tubes. After incubation at 37 °C for 2 h, the supernatant was obtained through centrifugation at 1500 rpm for 5 min and transferred to a 96-well plate. The absorbance at 540 nm was measured by a Multiskan FC microplate photometer (Thermo). RBCs in normal saline and deionized water were used as a negative control and a positive control, respectively. The following formula was used to calculate the hemolysis percentage:

$$Hemolysis\ (\%) = \frac{Sample\ absorbance\ -\ Negative\ control\ absorbance}{Positive\ control\ absorbance\ -\ Negative\ control\ absorbance} \times 100$$

Real-time imaging of *β*-gal in mice

Female BALB/c nude mice (6 weeks old) were obtained from the Animal Center of Xiangya Medical School, Central South University. All animal procedures were carried out under the guidelines approved by the Research Ethics Committee of the Xiangya School of Pharmaceutical Sciences, Central South University, China. To establish the tumor model, 200 μL PBS containing SKOV3 cells (1×10^6) were injected subcutaneously into the assigned site of nude mice. After 3 weeks, the tumor grew about 70 mm^3 and the tumor-bearing mice were randomly divided into two groups. In the experimental group, probe **OELyso-Gal** (100 μM, 100 μL PBS containing 20% DMSO) was intratumorally injected into the tumor site. In the inhibitor group, the tumor was pretreated with *D*-galactose (1 mM) for 2 h and then treated with **OELyso-Gal**. The *in vivo* fluorescence images were obtained at different times (10, 30, 60, 120 min) after treatment using the IVIS spectral imaging system. The fluorescence signal was collected at 650 nm with 570 nm as the excitation wavelength.

Tumour tissue imaging

The slices were prepared from the tumors of 3-week inoculated nude mice and then were sectioned at 400 μm thickness using a vibrating-blade microtome. The slices were incubated with **OELyso-Gal** (10 μM) or **OEN-Gal** (10 μM), respectively, in PBS buffer (10 mM, pH = 7.4, containing 30% DMSO) for 1 h at 37 °C. After washing with PBS three times, the tissues were transferred to the glass-bottomed dishes. The confocal fluorescence images were acquired at NIR channels (620-680 nm) upon excitation at 594 nm.

Table

Table S1. Comprehensive comparisons of OELyso-Gal and other reported probes.

Structure	$\lambda_{ex}/\lambda_{em}$ (nm)	K_m[a]	Response time[b]	LOD[c]	Lysosome-targeting	Imaging application	Ref.
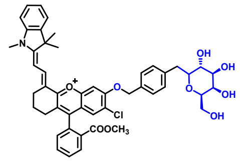	700/730	1.84 μM	5 min (0.5 U/mL)	2.0×10^{-3} U/mL	-	Breast cancer imaging and surgical resection navigation	2
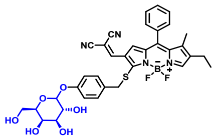	651/727	34.6 μM	-	1.4×10^{-2} U/mL	-	Senescent vascular cells in atherosclerotic mice	3
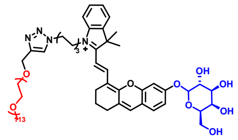	688/720	48.3 μM	40 min (0.6 U/mL)	-	-	Imaging-guided therapy of SKOV3 tumor	4
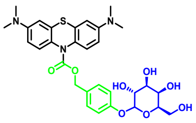	660/690	4.32 μM	10 min (0.5 U/mL)	1.56×10^{-4} U/mL	-	Detection and elimination of senescent cells	5
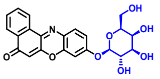	575/642	2.23 μM	50 min (1 U/mL)	9.6×10^{-2} U/mL	-	Diagnosis and resection of ovarian cancer in vivo	6
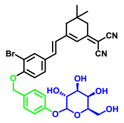	500/670	6.73 μM	13 min (1 U/mL)	5.0×10^{-3} U/mL	-	Imaging of senescent and cancer cells	7
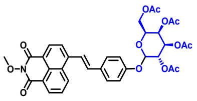	488/550	-	-	-	-	In vivo tracking of cellular senescence	8
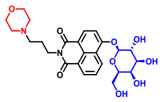	360/460; 460/560	-	30 min (150 U/L)	4.0×10^{-5} U/mL	Yes	Imaging of ovarian cancer cells	9
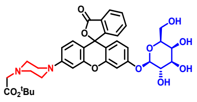	495/545	28.0 μM	30 min (0.1 U)	4.19×10^{-7} U/mL	Yes	Monitoring of vascular cell senescence	10
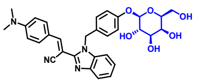	450/565	78.75 μM	30 min (10 U)	3.6×10^{-2} U/mL	Yes	Imaging of ovarian cancer cells	11
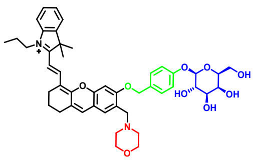	690/725	-	25 min (3 U/mL)	2.2×10^{-2} U/mL	Yes	Imaging of ovarian cancer cells	12

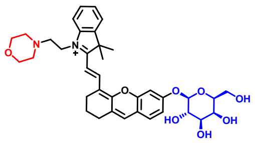	680/710	68.37 μM	250 s (10 U/mL)	1.2×10^{-2} U/mL	Yes	Imaging of senescent cells and ovarian cancer cells	13
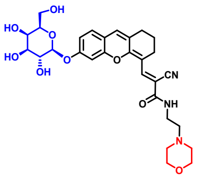	610/641	19.06 μM	20 min (1.2 U/mL)	3.2×10^{-3} U/mL	Yes	Imaging of ovarian cancer in vivo	This work

a, Km, the enzyme kinetic parameter Michaelis-Menten constant, which represents the affinity of probe to the enzyme. b, Response time, the time it takes for the probe to reach a plateau in fluorescence signal change. c, LOD, the limit of detection.

Figures

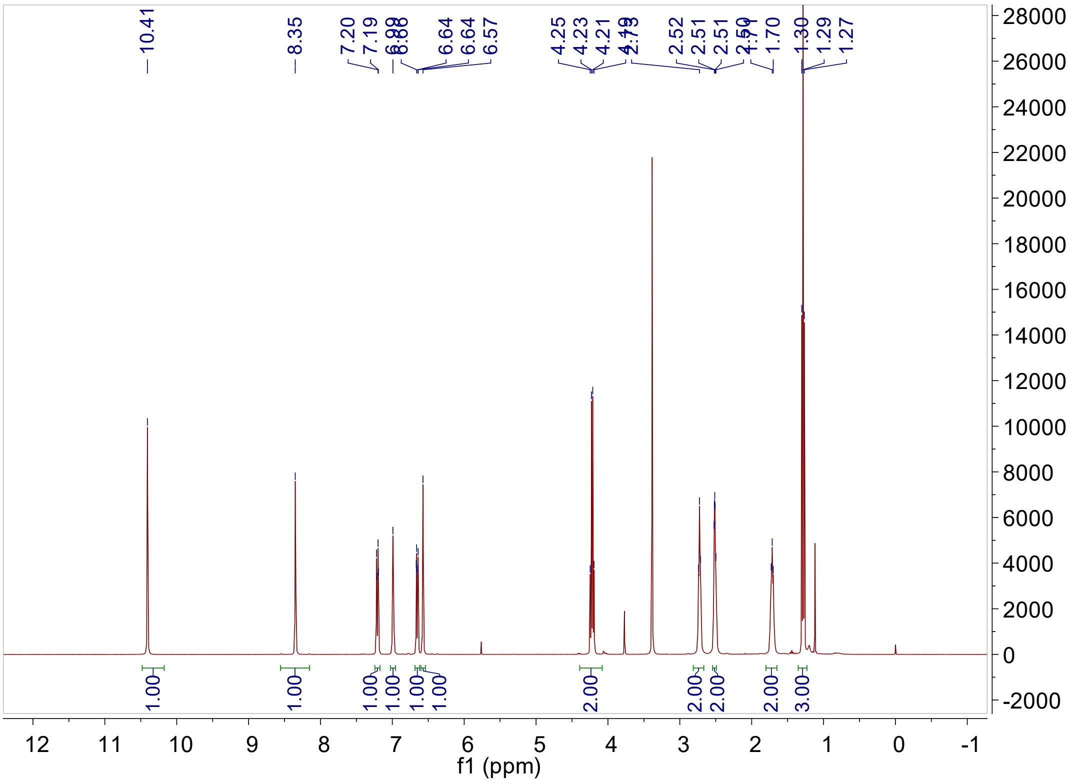

Fig S1. ^{1}H-NMR spectra of **OEE-OH.**

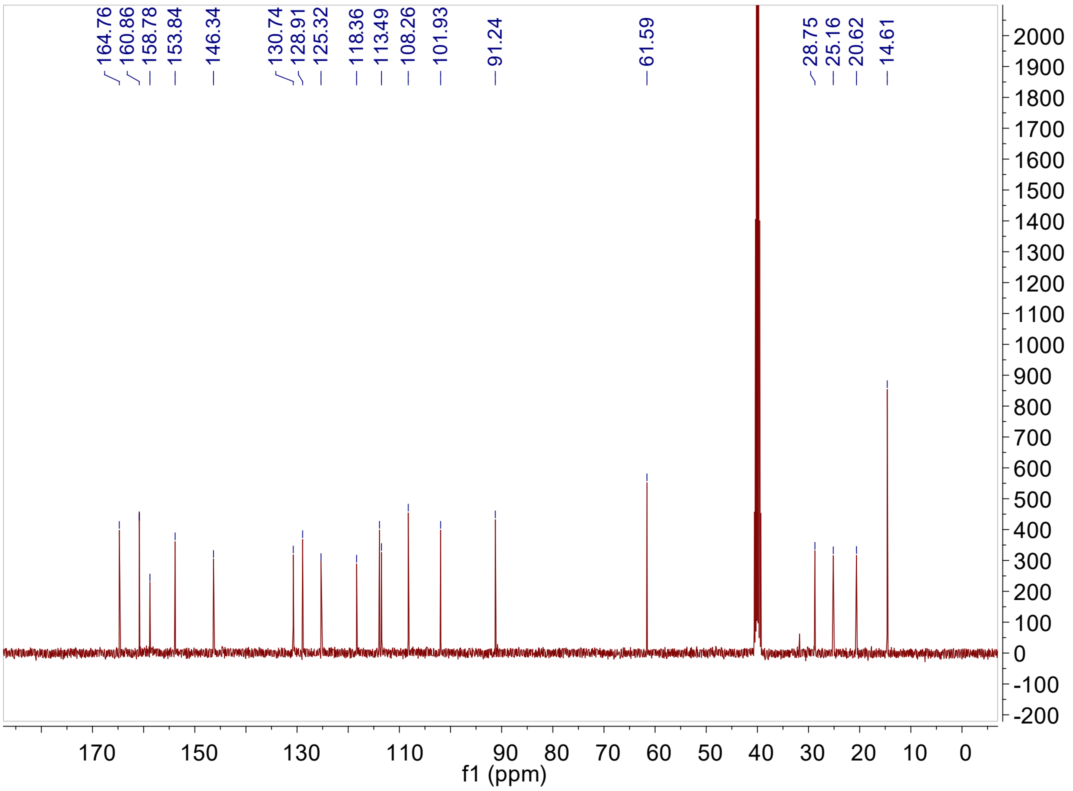

Fig S2. ^{13}C-NMR spectra of **OEE-OH**.

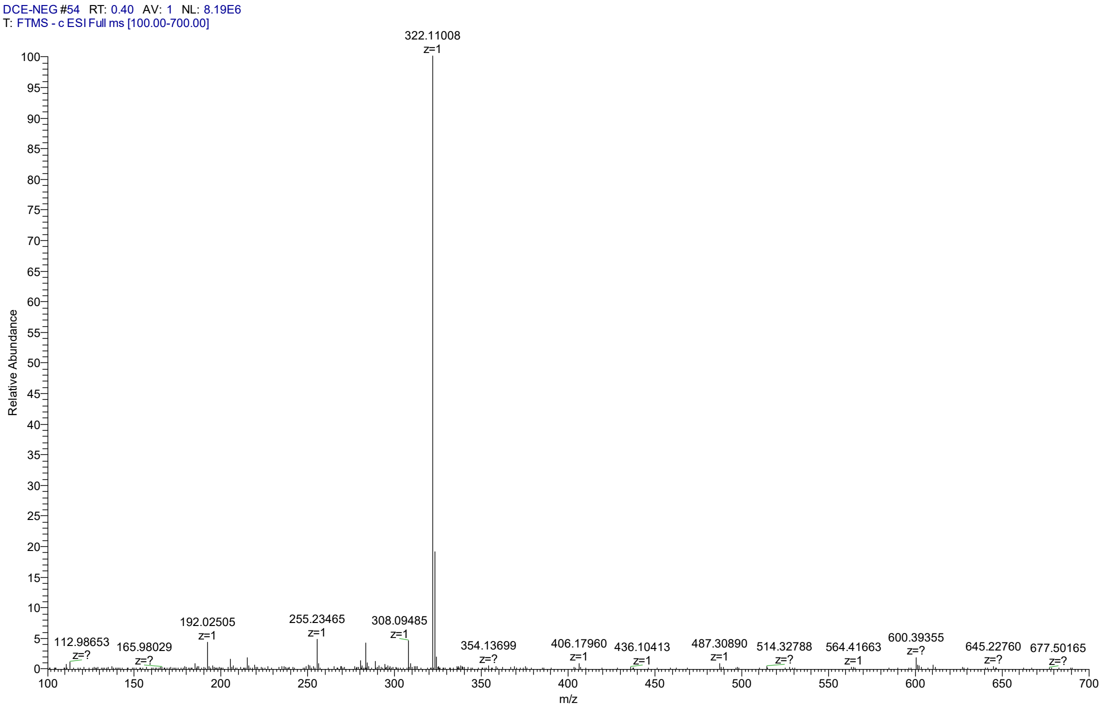

Fig S3. HRMS spectra of **OEE-OH**.

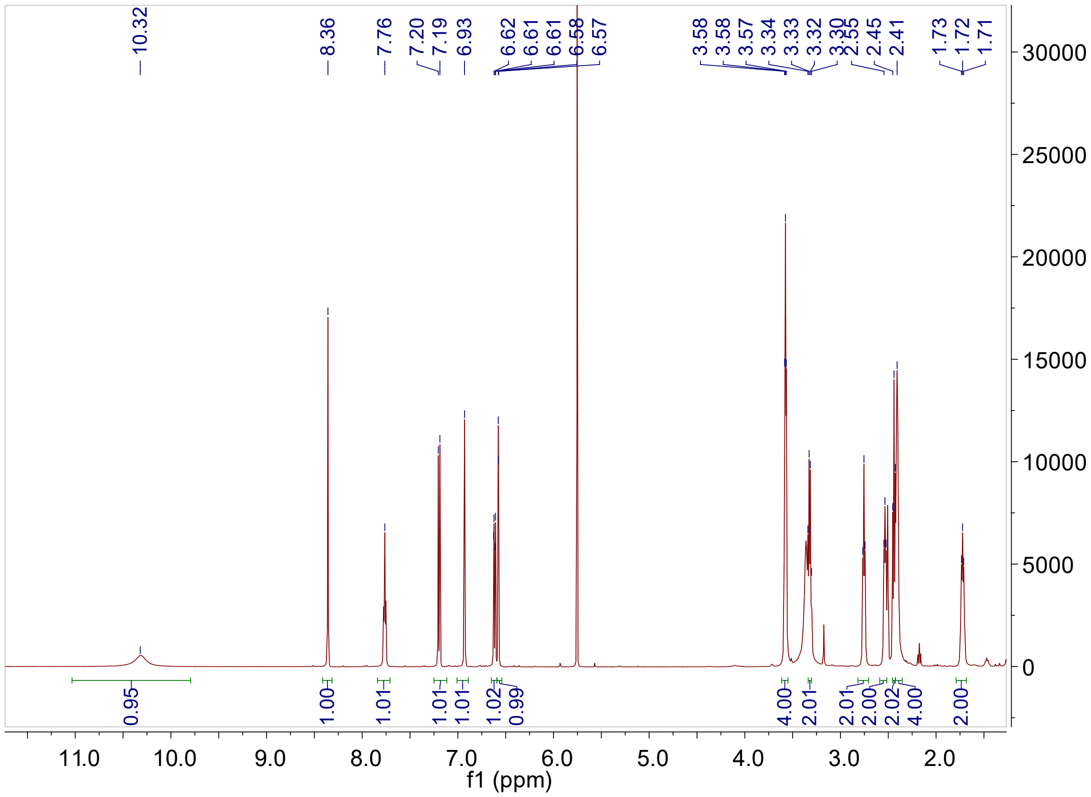

Fig S4. ^{1}H-NMR spectra of **OELyso-OH.**

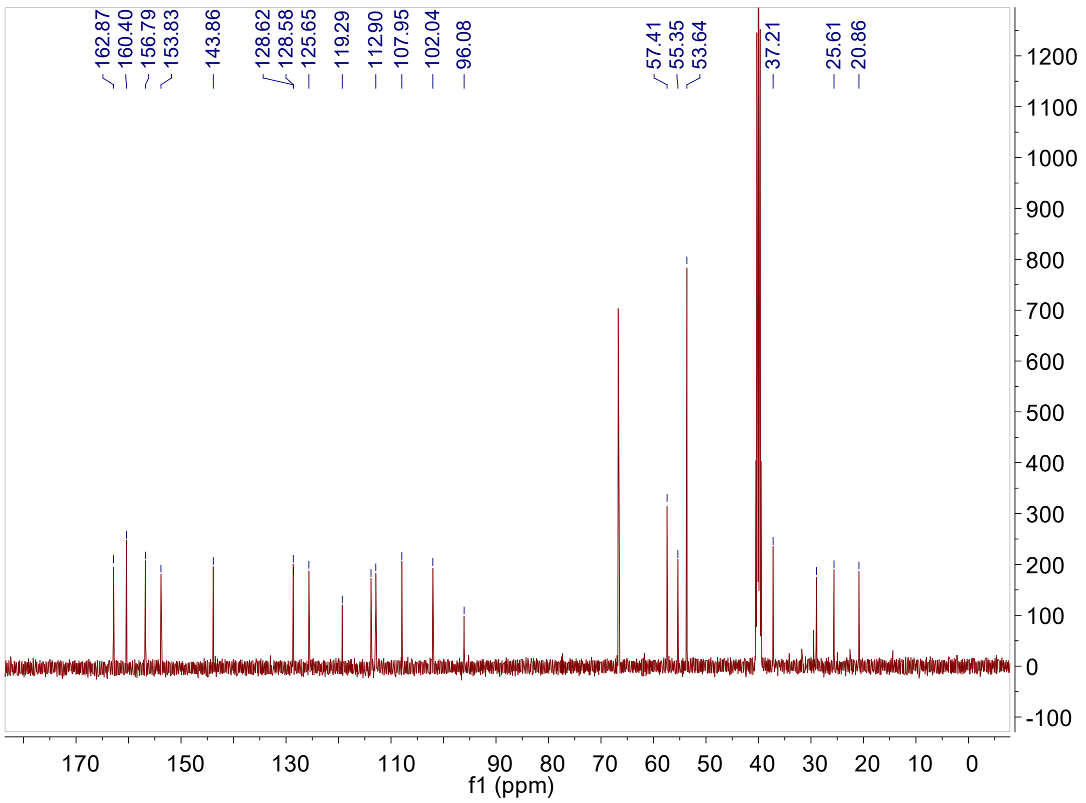

Fig S5. 13C-NMR spectra of **OELyso-OH**.

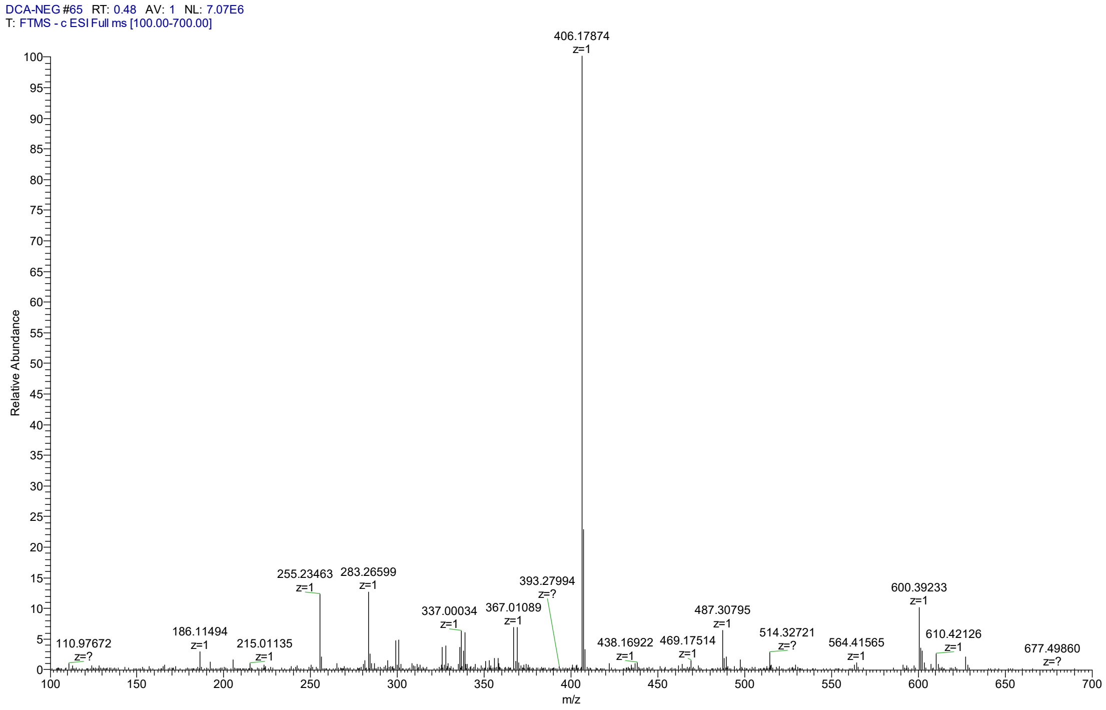

Fig S6. HRMS spectra of **OELyso-OH**.

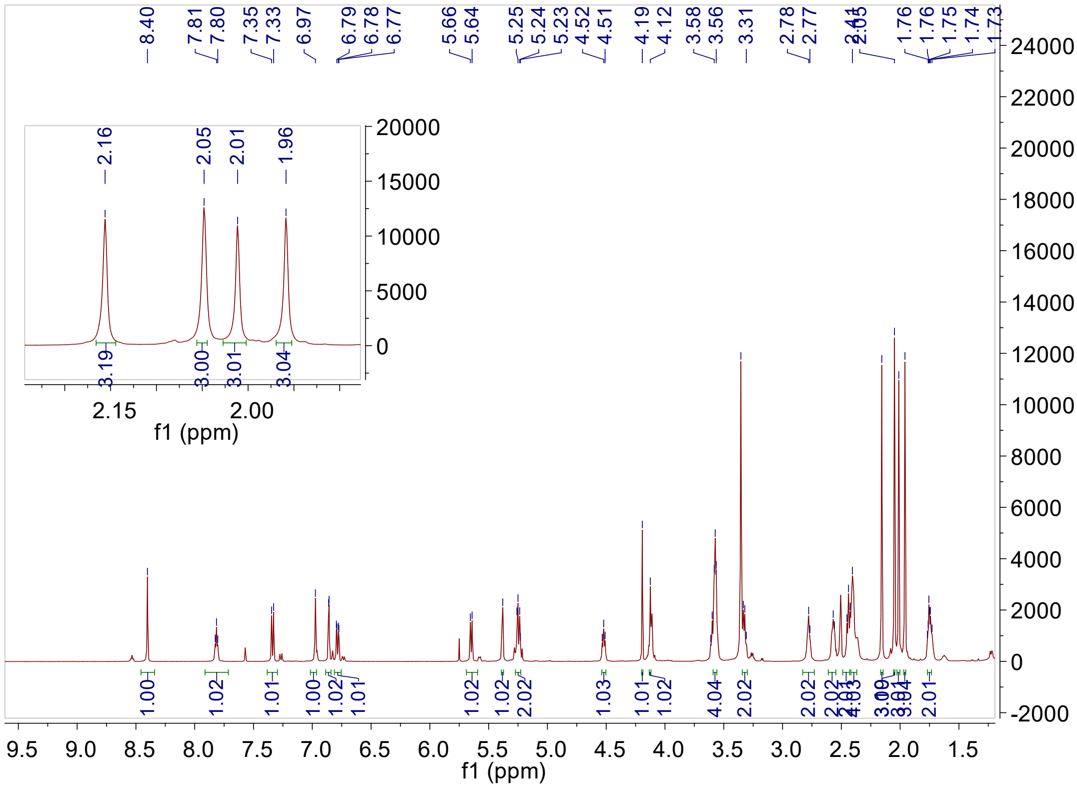

Fig S7. ^{1}H-NMR spectra of **OELyso-AcGal.**

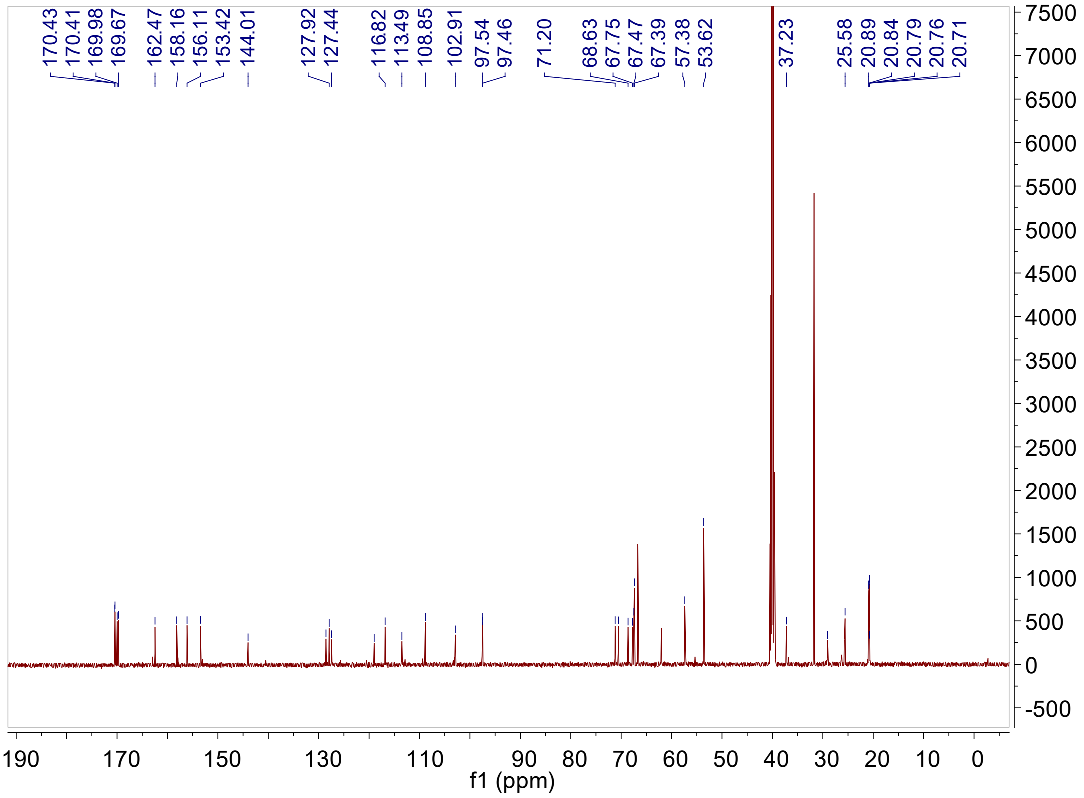

Fig S8. 13C-NMR spectra of **OELyso-AcGal**.

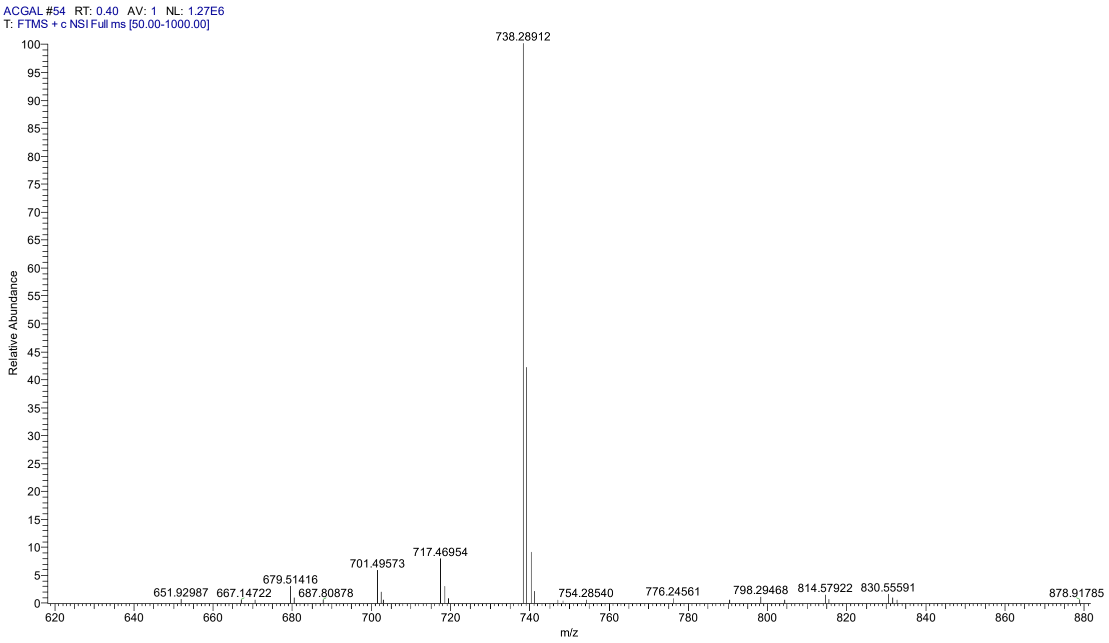

Fig S9. HRMS spectra of **OELyso-AcGal**.

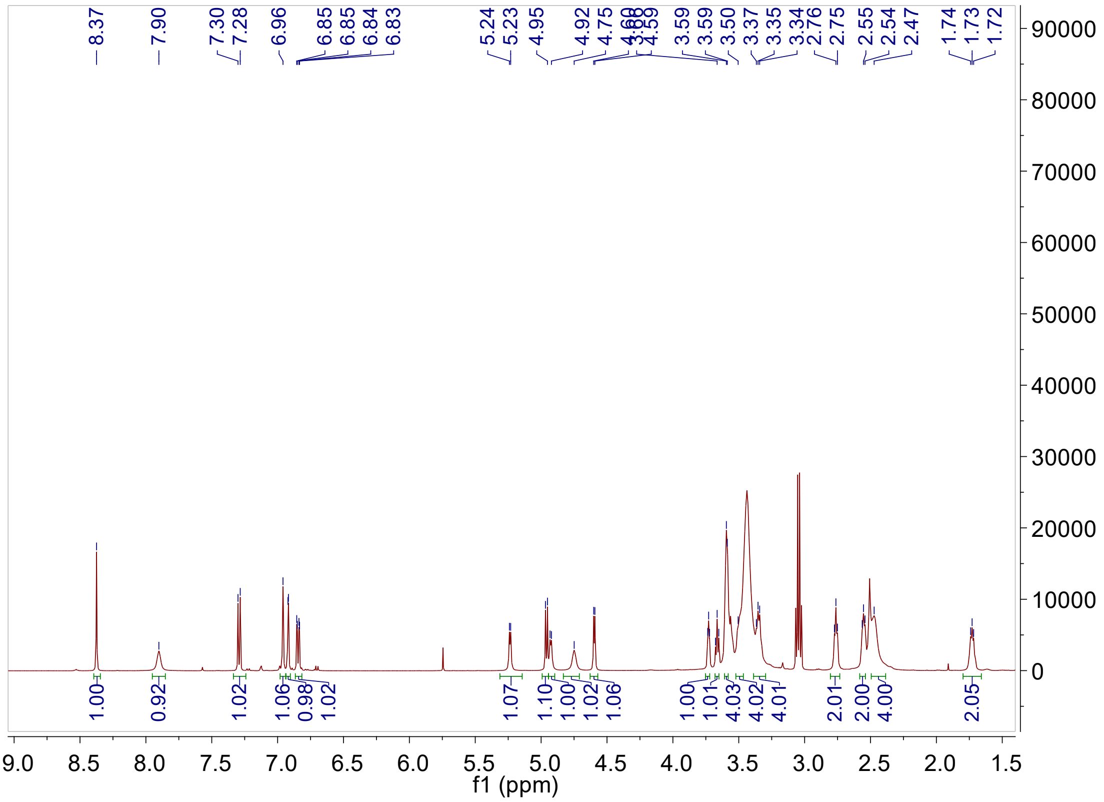

Fig S10. ^{1}H-NMR spectra of **OELyso-Gal.**

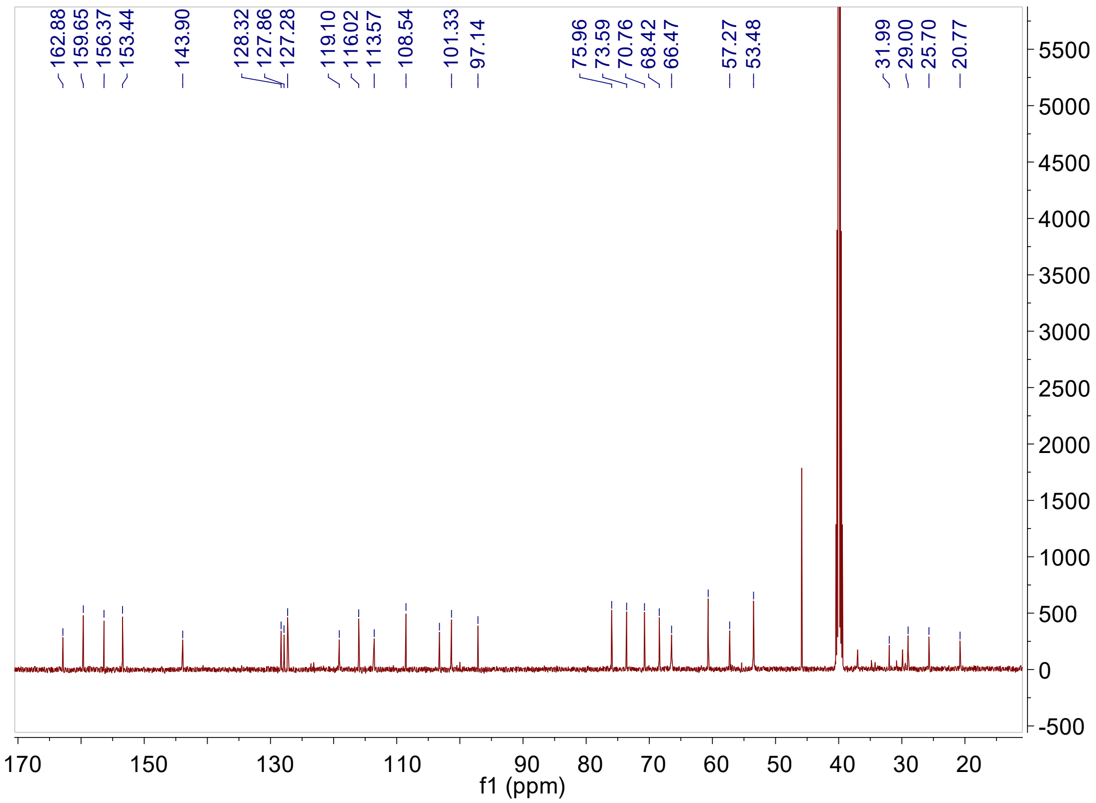

Fig S11. 13C-NMR spectra of **OELyso-Gal**.

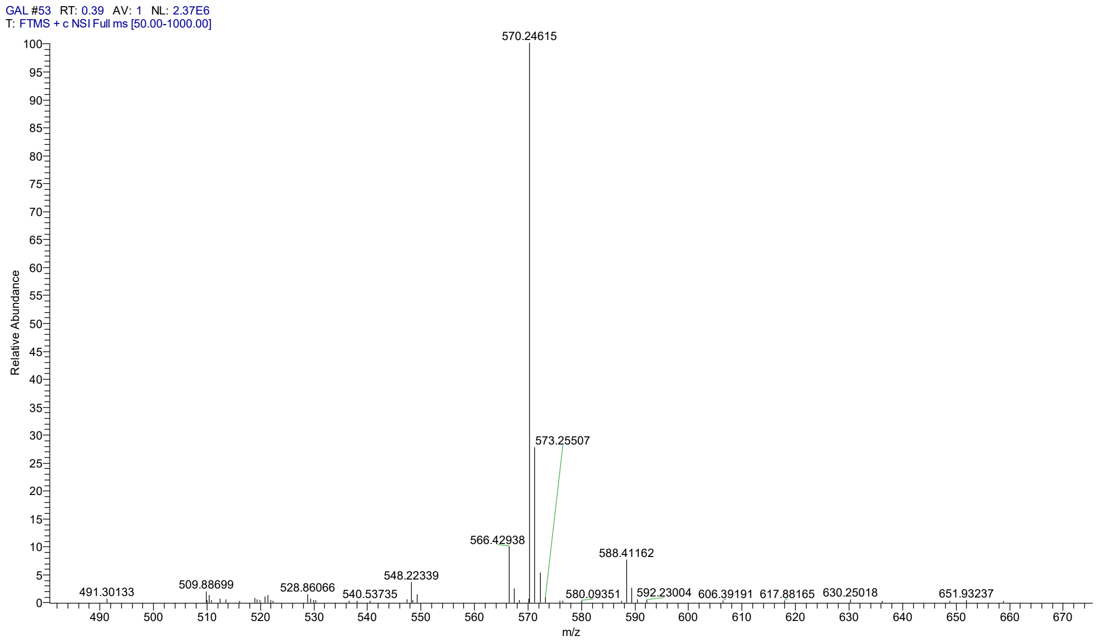

Fig S12. HRMS spectra of **OELyso-Gal**.

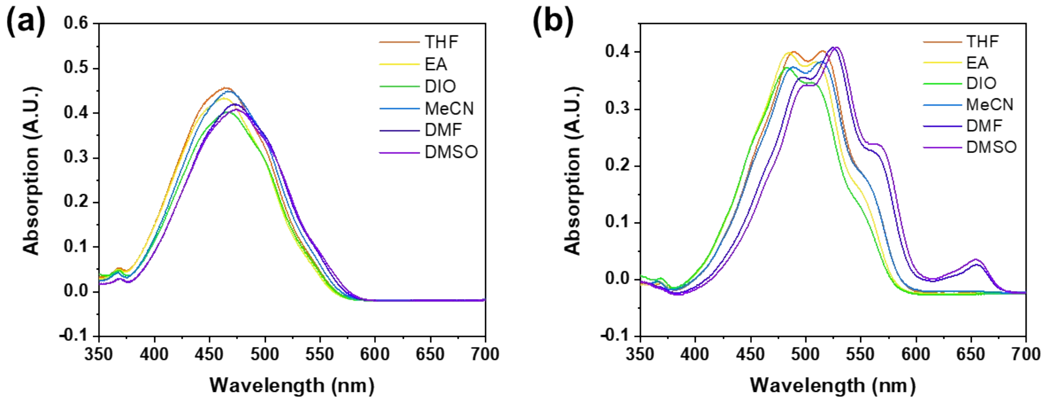

Fig S13. Solvatochromic absorption spectra of **OELyso-OH** and **OEN-OH** in different polar aprotic solvents.

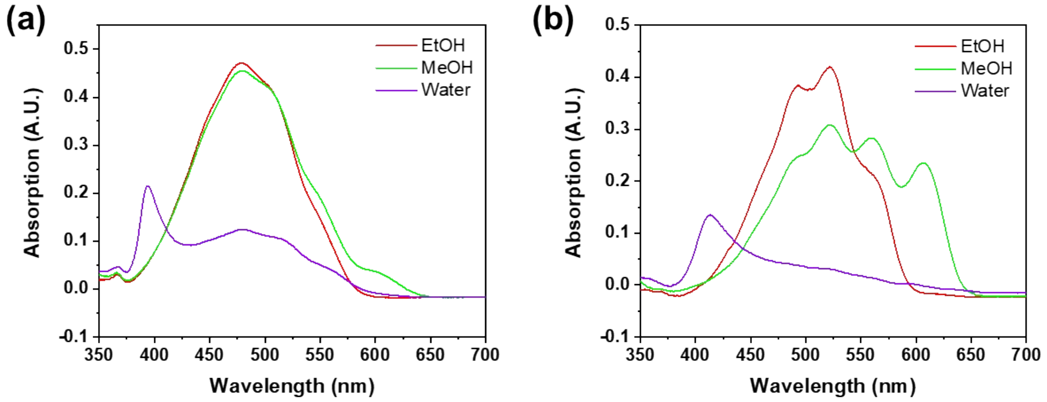

Fig S14. Solvatochromic absorption spectra of **OELyso-OH** and **OEN-OH** in different polar protic solvents.

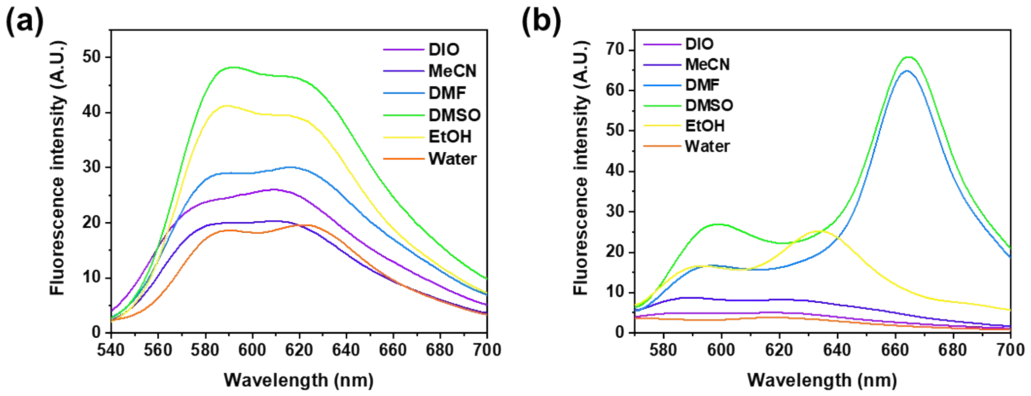

Fig S15. Solvatochromic emission spectra of **OELyso-OH** and **OEN-OH** in different solvents. λ_{ex} = 500 nm.

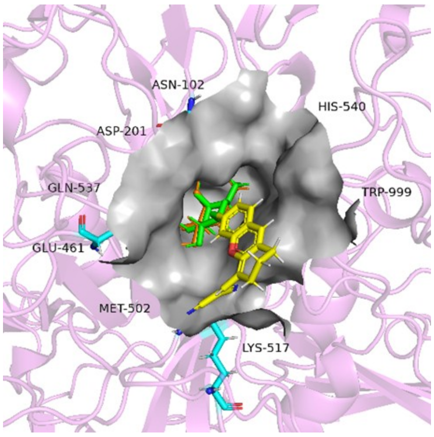

Fig S16. Molecular docking simulation of the binding mode between **OEN-Gal** (in yellow) and *β*-gal (ligand of galactose was displayed in orange).

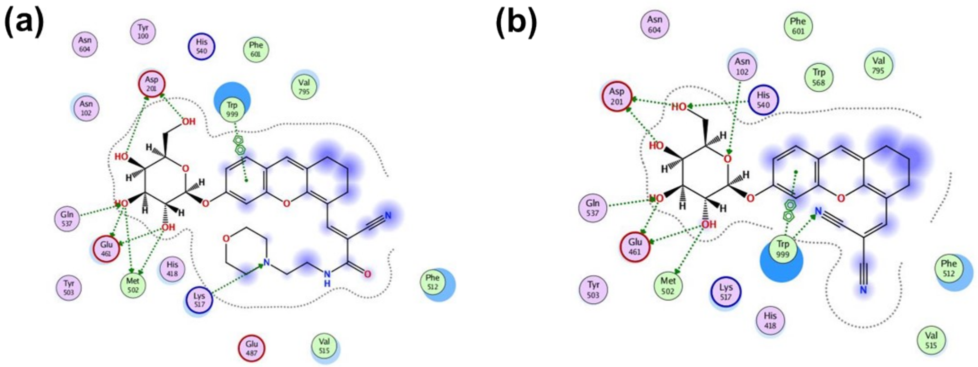

Fig S17. Interaction diagram between probe **OELyso-Gal** (a) and **OEN-Gal** (b) and the enzyme of *β*-gal.

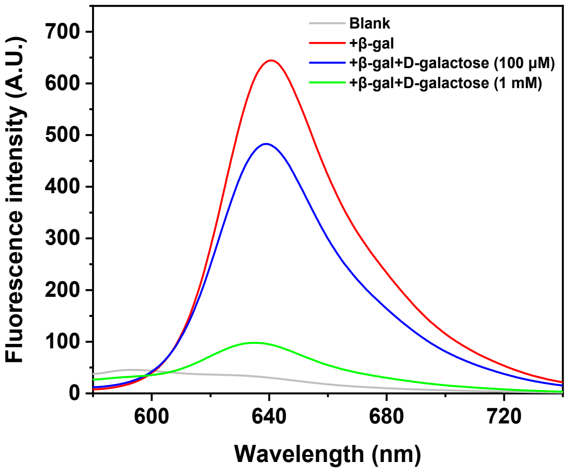

Fig. S18. Competitive inhibition of **OELyso-Gal** (10 μM) after incubation with *β*-gal (1.2 U/mL) for 30 min in the presence of different concentration of inhibitors (0, 100 μM, and 1 mM) in solution (PBS/DMSO = 7:3, V/V, pH 7.4, 37 °C). λ_{ex} = 560 nm.

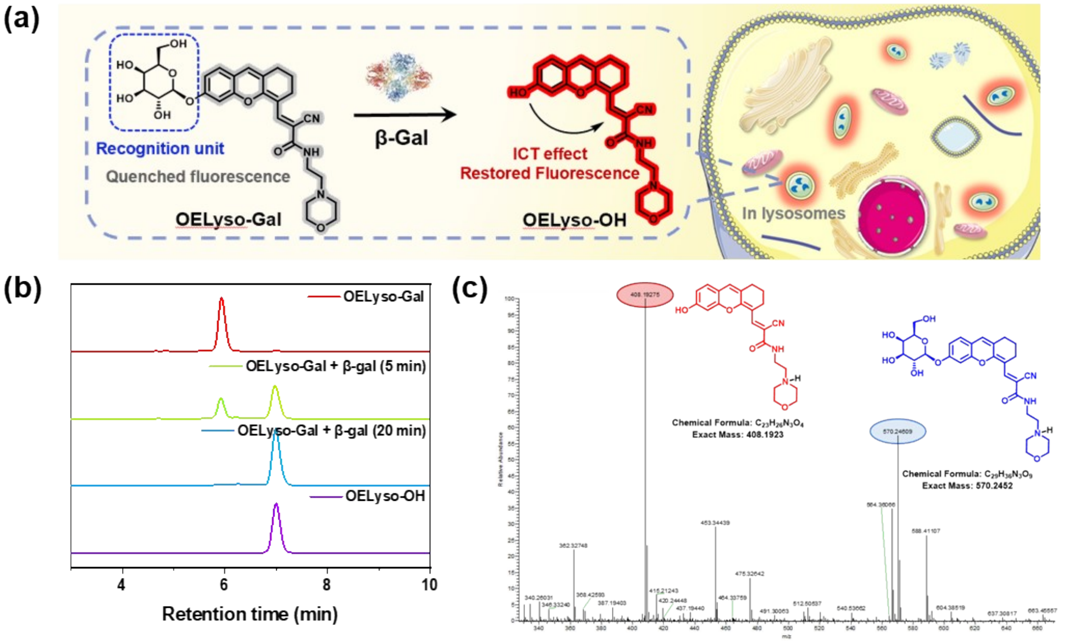

Fig S19. (a) Proposed sensing mechanism of **OELyso-Gal** for specifically imagingβ-gal in lysosomes. (b) HPLC analysis of **OELyso-Gal, OELyso-Gal** incubated with β-gal for 5 min or 20 min, and **OELyso-OH**. (c) HRMS analysis of **OELyso-Gal** after incubation with β-gal (1.2 U/mL) for 30 min in solution (PBS/DMSO = 7:3, V/V, pH 7.4, 37 °C).

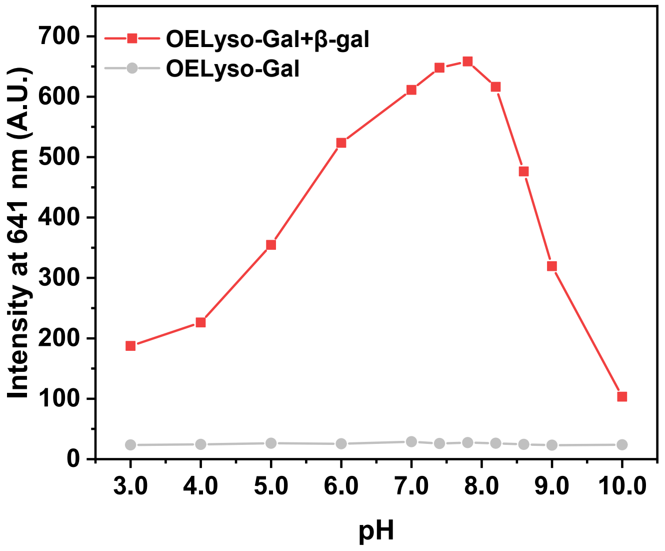

Fig. S20. Fluorescence intensities of **OELyso-Gal** (10 μM) at 641 nm after incubation with β-gal (1.2 U/mL) for 30 min in solution (PBS/DMSO = 7:3, V/V, pH 7.4, 37 °C). λ_{ex} = 560 nm.

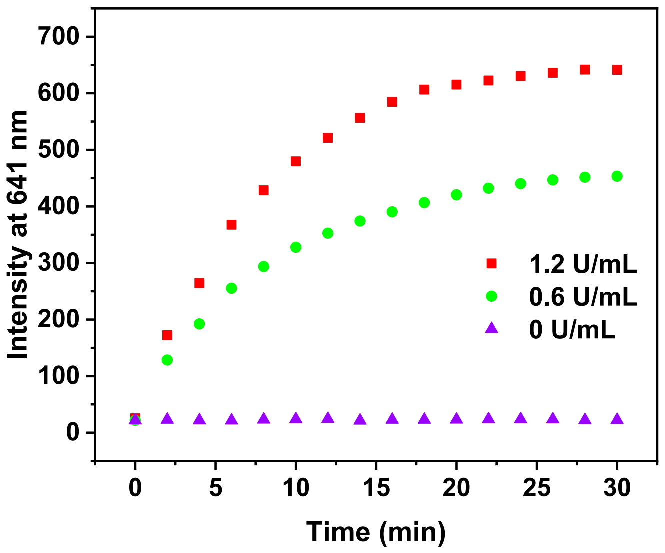

Fig. S21. Time-dependent fluorescence intensity of **OELyso-Gal** (10 μM) at 641 nm in the presence of *β*-gal (0 U/mL, 0.6 U/mL, 1.2 U/mL) in solution (PBS/DMSO = 7:3, V/V, pH 7.4, 37 °C). λ_{ex} = 560 nm.

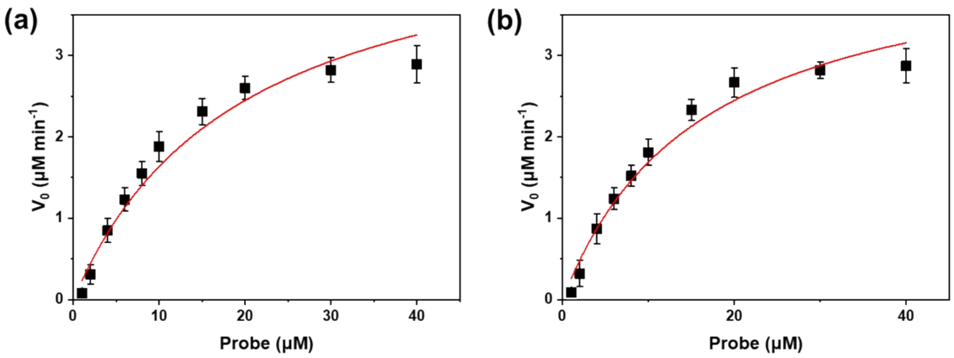

Fig. S22. Nonlinear fitting of Michaelis-Menten plot of V_0 as a function of **OELyso-Gal** (a) and **OEN-Gal** (b) concentration (2-80 μM) in presence of *β*-gal (1.2 U/mL) in solution (PBS/DMSO = 7:3, V/V, pH 7.4, 37 °C).

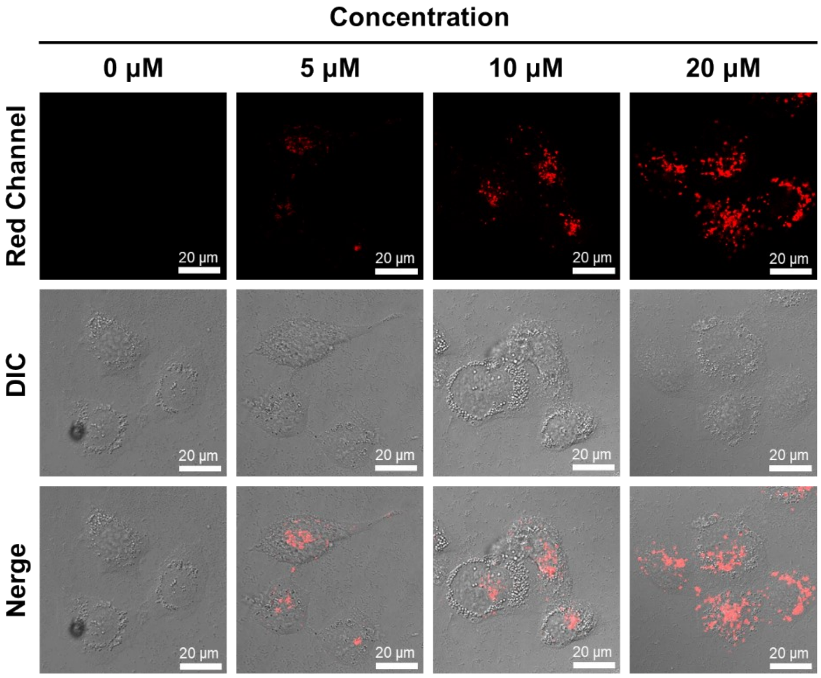

Fig S23. Concentration-dependent fluorescence images and bright-field images of **OELyso-Gal** (10 μM)-stained SKOV-3 cells. λ_{ex} = 605 nm, λ_{em} = 620-680 nm. Scale bar: 20 μm.

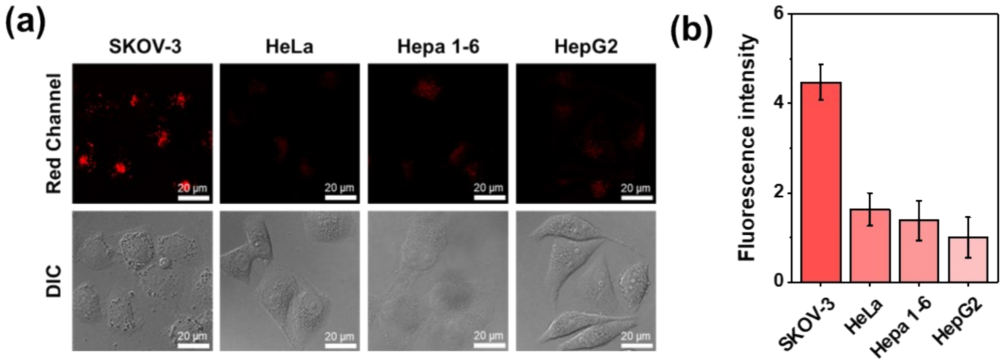

Fig S24. Fluorescence images (a) and relative fluorescent intensity (b) of **OELyso-Gal** (10 μM)-staining SKOV-3, HeLa, Hepa 1-6, and HepG2 cells. λ_{ex} = 605 nm, λ_{em} = 620-680 nm. Scale bar: 20 μm.

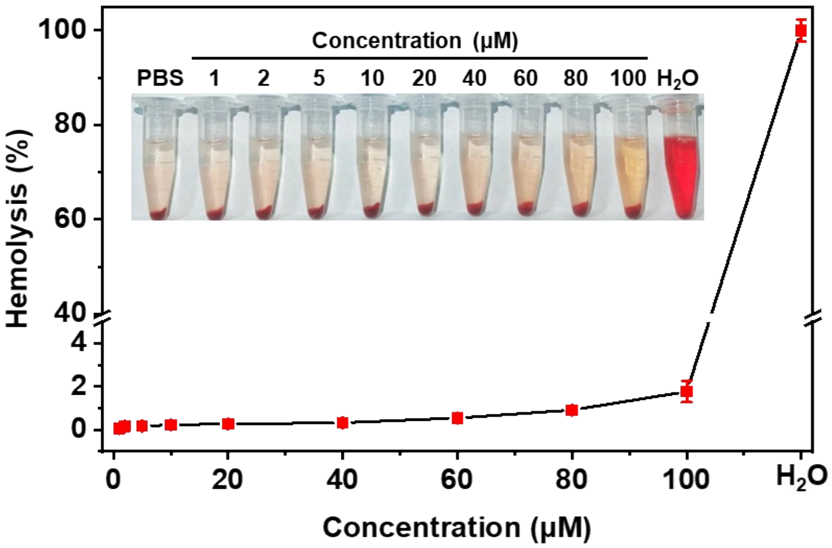

Fig S25. Hemolysis assay of **OELyso-Gal** (1-100 μM).

References

1 Z. Tang and P. Zhou, *J. Phys. Chem. B*, 2020, **124**, 3400-3407.

2 Q. Wu, Q. H. Zhou, W. Li, T. B. Ren, X. B. Zhang and L. Yuan, *ACS Sens*, 2022, **7**, 3829-3837.

3 J. A. Chen, W. Guo, Z. Wang, N. Sun, H. Pan, J. Tan, Z. Ouyang, W. Fu, Y. Wang, W. Hu and X. Gu, *Anal. Chem.*, 2020, **92**, 12613-12621.

4 X. Zhen, J. Zhang, J. Huang, C. Xie, Q. Miao and K. Pu, *Angew. Chem. Int. Ed. Engl.*, 2018, **57**, 7804-7808.

5 L. Yang, G. Liu, Q. Chen, Y. Wan, Z. Liu, J. Zhang, C. Huang, Z. Xu, S. Li, C. S. Lee, L. Zhang and H. Sun, *Anal. Chem.*, 2022, **94**, 5425-5431.

6 L. Xu, H. Chu, D. Gao, Q. Wu, Y. Sun, Z. Wang, P. Ma and D. Song, *Anal. Chem.*, 2023, **95**, 2949-2957.

7 S. Chen, L. Wang, X. Ma, Y. Wu and S. Hou, *Sens. Actuat. B Chem.*, 2022, **367**, 132061-132071.

8 B. Lozano-Torres, J. F. Blandez, I. Galiana, J. A. Lopez-Dominguez, M. Rovira, M. Paez-Ribes, E. Gonzalez-Gualda, D. Munoz-Espin, M. Serrano, F. Sancenon and R. Martinez-Manez, *Anal. Chem.*, 2021, **93**, 3052-3060.

9 J. Huang, N. Li, Q. Wang, Y. Gu and P. Wang, *Sens. Actuat. B Chem.*, 2017, **246**, 833-839.

10 E.-J. Kim, A. Podder, M. Maiti, J. M. Lee, B. G. Chung and S. Bhuniya, *Sens. Actuat. B Chem.*, 2018, **274**, 194-200.

11 Y. Li, L. Ning, F. Yuan, T. Zhang, J. Zhang, Z. Xu and X. F. Yang, *Anal. Chem.*, 2020, **92**, 5733-5740.

12 X. Li, Y. Pan, H. Chen, Y. Duan, S. Zhou, W. Wu, S. Wang and B. Liu, *Anal. Chem.*, 2020, **92**, 5772-5779.

13 H. Pan, X. Chai and J. Zhang, *Chin. Chem. Lett.*, 2023, **34**, 108321-108324.
